# Pharmacological characterization and nephroprotective potential of venoms from three egyptian scorpion species

**DOI:** 10.3389/fphar.2026.1795320

**Published:** 2026-08-28

**Authors:** Alhussin M. A. Megaly, Masahiro Miyashita, Abdulaziz R. Alqahtani, Mahmoud Ashry, Mohammed Abdel-Wahab

**Affiliations:** 1 Zoology Department, Faculty of Science, Al-Azhar University, Assiut, Egypt; 2 Division of Applied Life Sciences, Graduate School of Agriculture, Kyoto University, Kyoto, Japan; 3 Department of Biology, College of Science, University of Bisha, Bisha, Saudi Arabia

**Keywords:** antioxidant mechanisms, drug-induced nephrotoxicity, inflammation, natural products pharmacology, paclitaxel, scorpion venom, venom peptides

## Abstract

**Introduction:**

Scorpion venoms are complex natural mixtures rich in bioactive molecules with emerging pharmacological relevance. Drug-induced kidney injury, particularly from chemotherapy agents like Taxol® (paclitaxel), remains a significant clinical challenge. This study investigates the molecular composition, enzymatic activities, and potential nephroprotective effects of venoms from three Egyptian scorpion species (Leiurus quinquestriatus, Buthacus leptochelys, and Scorpio maurus palmatus) against Taxol®-induced kidney injury.

**Methods:**

Comprehensive molecular mass profiling of the three venoms was performed using liquid chromatography–mass spectrometry (LC-MS). Enzymatic characterization included assays for hyaluronidase, phospholipase A2 (PLA2), hemolytic, and antibacterial activities. The disulfide-bonded versus non-disulfide-bonded peptide (NDBP) ratios were also determined. To evaluate nephroprotective efficacy, the venoms were administered in an in vivo model of Taxol®-induced nephrotoxicity. Renal antioxidant defenses, oxidative stress markers, inflammatory responses, and histopathological architecture were assessed.

**Results:**

LC-MS revealed hundreds of venom components within the 500–9,000 Da range, indicating a high diversity of low-molecular-weight peptides. All venoms exhibited notable hyaluronidase activity, whereas PLA2 activity varied markedly: absent in L. quinquestriatus, low in B. leptochelys, and pronounced in S. m. palmatus. Minor hemolytic activity was observed only in B. leptochelys, and all venoms demonstrated weak antibacterial effects. The ratio of disulfide-bonded to non-disulfide-bonded peptides ranged from 7.5% NDBPs in L. quinquestriatus to 48% in S. m. palmatus. Despite these biochemical differences, all three venoms significantly attenuated Taxol-induced nephrotoxicity, as evidenced by restored renal antioxidant defenses, suppressed oxidative stress and inflammatory responses, and improved renal histopathological architecture.

**Discussion:**

The inverse correlation between PLA2 and hyaluronidase activities, coupled with striking differences in peptide composition, exemplifies a biochemical trade-off in venom resource allocation—highlighting how distinct molecular solutions evolve to meet similar ecological demands. From a pharmacological perspective, the comparable nephroprotective efficacy across species suggests that scorpion venoms contain bioactive constituents capable of modulating key molecular pathways involved in drug-induced renal injury. In a pharmacoepidemiologic context, these findings highlight the potential of venom-derived compounds as adjunctive or protective agents to mitigate chemotherapy-associated nephrotoxicity, supporting further investigation into their safety, efficacy, and translational applicability.

## 1 Introduction

Scorpion venom is a complex mixture of bioactive components, forming a sophisticated biochemical arsenal that includes a diverse array of molecules. These components play crucial roles in immobilizing prey and defending against predators, in which bioactive components are peptides and proteins (Quintero-Hernández et al., 2013). The composition of scorpion venom varies with species, geographical location, age, and sex, resulting in a wide range of biological activities. The major bioactive components of scorpion venom are disulfide-bridged peptides (DBPs) that target ion channels (sodium, potassium, and calcium). These affect nerve and muscle functions to cause paralysis and pain in prey and predators (Possani et al., 1999). Most insecticidal toxins exhibit highly selective actions, which is attributed to their specific action on insect ion channels (Valdez-Velazquéz et al., 2016; Yoshimoto et al., 2019). The venom also contains non-disulfide-bridged peptides (NDBPs) with potent cytolytic property (Almaaytah and Albalas, 2014). Antimicrobial activity of the venom is primarily due to NDBPs by disrupting bacterial cell membranes. This action mechanism makes them less susceptible to resistance development compared to conventional antibiotics (Ortiz et al., 2015). Furthermore, some of them have potential against antibiotic-resistant strains of bacteria (Abdallnasser Amen et al., 2025; Megaly et al., 2025; Harrison et al., 2014; Xia et al., 2024). Enzymes such as hyaluronidases and phospholipases in the venom are known not only to contribute to local effects but also to enhance the spread of venom components (Vejayan et al., 2014; Díaz-García α et al., 2015; Mendoza-Tobar et al., 2024). Small molecules such as amino acids, biogenic amines (like histamine and serotonin), nucleotides, and inorganic salts also contribute to the overall effects of venom (Harrison et al., 2014). Other reported biological activities include antiviral, antifungal, anticancer, anti-inflammatory, and immunomodulatory effects (El-Bitar et al., 2015; Yoshimoto et al., 2019; Dal Belo et al., 2025). Thus, the components of scorpion venom provide important insight for development of pharmaceuticals.

Considerable attention has been given to the venoms of the Egyptian scorpions due to their potent neurotoxic properties. *Leiurus quinquestriatus* (Buthidae), known as the “deathstalker,” is among the most medically important scorpions, which possess venom with potent insect and mammalian neurotoxicity. It also contains peptides that show antimicrobial and anticancer activities (Al-Asmari et al., 2016; Trop et al., 2021; El-Qassas et al., 2024). *Buthacus leptochelys* (Buthidae), native to North Africa and the Middle East, produces venom rich in neurotoxins and antimicrobial peptides. The venom contains insecticidal peptides like Bl-1, which has a structure similar to alpha-like toxins (Yoshimoto et al., 2019). Recent research has identified peptides with significant activity against both Gram-positive and Gram-negative bacteria, including methicillin-resistant *Staphylococcus aureus* (MRSA) and other multidrug-resistant pathogens (Rabea et al., 2025). Also, some components have shown synergistic effects with conventional antibiotics, enhancing their efficacy against resistant strains (Zhao et al., 2009). *Scorpio maurus palmatus* (Scorpionidae) venom includes maurotoxin, a potassium channel blocker, and maurocalcin, a calcium channel modulator, suggesting its potential for discovering unknown neurotoxic peptides (Castle et al., 2003; Bergeron and Bingham, 2012; Abdel-Rahman et al., 2013; Nguyen et al., 2022). Unlike Buthidae species, the venom of *S. maurus palmatus* is rich in defensin-like peptides with broad-spectrum antibacterial and antifungal effects. These peptides exhibit membrane-permeabilizing activity, making them effective against drug-resistant fungal pathogens (Moerman et al., 2002). However, the enzymatic activity of the venoms from these scorpion species has been poorly evaluated.

In this study, we performed detailed molecular mass profiling of the components of the venom of *B. leptochelys*, *L. quinquestriatus*, and *S. maurus palmatus*, as well as their biochemical and functional analyses. Emphasis was placed on their enzymatic activity, such as hyaluronidases and phospholipases, which play a crucial role in toxicity and spread of components of scorpion venom. Comparison of the chemical and functional properties of the venoms between these scorpion species will facilitate our understanding of their toxicity and potential biomedical applications.

## 2 Materials and methods

### 2.1 Extraction of venom from scorpions

Specimens of *B. leptochelys*, *S. m. palmatus*, and *L. quinquestriatus* were collected from different sites in Egypt. For each species, venom was obtained from at least 10 individuals. Venom was extracted from the venom glands using fine forceps and then manually mixed in distilled water. All procedures were performed on ice to preserve enzymatic activity. The collected venoms were then centrifuged at 14,000 rpm for 10 min at 4 °C. The supernatants were collected, freeze-dried, and stored at (−20 °C) until use.

#### 2.1.1 Selection of dose and safety assessment

Prior to the *in vivo* nephroprotection study, acute toxicity tests were conducted in male albino rats (n = 5, 150–200 g) to determine safe, sub-lethal doses of each crude venom. Venom solutions were administered intraperitoneally at escalating doses (1, 5, 10, 20, 50, 100, and 200 μg/kg). Animals were observed for 6 h continuously and then daily for 7 days for signs of toxicity (irritability, salivation, dyspnea, convulsions, or death).

No toxic effects were observed at doses ≤10 μg/kg for any venom. The maximum tolerated dose (MTD) was therefore established as 10 μg/kg for each species. The LD50 could not be determined as no mortality occurred up to 200 μg/kg. Based on these findings, a dose of 10 μg/kg/day was selected for the 4-week nephroprotection study. This dose is well below reported LD50 values for related Buthidae (0.five to five mg/kg) and Scorpionidae (>10 mg/kg) venoms (Abdel-Rahman et al., 2013).

### 2.2 Electrophoresis

Crude venoms (30 µg in 12 µL) were analyzed using sodium dodecyl sulfate–polyacrylamide gel electrophoresis (SDS–PAGE) on 15% (w/v) gels under nonreducing conditions, following the method of Laemmli (1970) (Laemmli, 1970). Electrophoresis was performed alongside a pre-stained protein ladder marker (range 10–245 kDa, nacalai tesque, Kyoto, Japan) for determination of molecular weight. The gels were subsequently stained with Coomassie Brilliant Blue G250 (1.25 g/L) and subsequently imaged. Protein band analysis was conducted using Gel Analyzer (GelAnalyzer 23.1.1 b y Istvan Lazar Jr, PhD and Istvan Lazar Sr, PhD, CSc) (Nakládal et al., 2025).

### 2.3 High-performance liquid chromatography

Crude venom samples (10 μg) were injected into a reversed-phase high-performance liquid chromatography (HPLC) column (Everest C18, 5 μm, 250 × 1.0 mm, Hichrom, Reading, UK). Solvent A (0.1% TFA in water) and solvent B (0.08% TFA in acetonitrile) were eluted through the column at a flow rate of 0.05 mL/min using a linear gradient of 5%–60% of solvent B over 55 min. Elution was monitored by ultraviolet absorbance at 215 nm.

### 2.4 Mass spectrometric analysis

The crude venom (2.0 µg) was analyzed by high-resolution LC/MS using an Orbitrap Exploris 240 instrument (Thermo Fisher Scientific) equipped with a heated electrospray ionization (HESI) source. Separation was achieved on a reversed-phase column (Grace Vydac Everest C18, 5 μm, 250 × 1.0 mm) with a linear gradient of 5%–50% acetonitrile (containing 0.1% formic acid) in 0.1% formic acid over 50 min at 0.05 mL/min. MS measurements were performed in positive mode using a nano-electrospray ion source under the following conditions: spray voltage 3500 V; sheath gas 35 arbitrary units; auxiliary gas seven arbitrary units; ion transfer tube temperature 320 °C; vaporizer temperature 275 °C; internal calibration m/z 391.28429 and 445.12003; resolution 60,000; scan range m/z 300–2000; maximum injection time 100 m; normalized AGC target 300%; RF lens 70%.

### 2.5 Alkylation and reduction of cysteine residues

Crude venom (100 μg) was dissolved in a buffer containing 130 mM NaHCO_3_ (pH 8.5), 2.7 M urea, and 35 mM dithiothreitol. The mixture was first incubated for 1 h at 50 °C, then it was treated with iodoacetamide, which was used at a final concentration of 125 mM, for another 1 h at 25 °C. The samples were analyzed by LC/MS directly without prior purification.

### 2.6 Hyaluronidase activity

Hyaluronidase activity within the venom samples was assessed by SDS–PAGE, following the methodology outlined in a prior study (Costal-Oliveira et al., 2012; Sutti et al., 2014; Megaly et al., 2025). To facilitate this analysis, hyaluronic acid was incorporated into a 10% resolving gel at a final concentration of 0.5 mg/mL. The crude venom was prepared by dissolving it in a 2× Laemmli buffer under non-reducing conditions. This buffer contained 4% SDS, 20% glycerol, 0.004% bromophenol blue, and 125 mM Tris-HCl (pH 6.8), with a venom concentration of 10 µg. Electrophoresis was carried out at room temperature under a constant voltage condition until the tracking dye had completely migrated to the gel’s lower edge. Upon completion of electrophoresis, the gel was subjected to two consecutive washes in 50 mL of 0.1 M sodium phosphate buffer (pH 5.8) containing 0.15 M NaCl and 5% Triton X-100. Each wash lasted for 1 h The gel was then transferred to 50 mL of the same phosphate buffer with a reduced Triton X-100 concentration of 0.05% for an additional 1 h. Subsequently, it was immersed for 10 min in 50 mL of phosphate buffer at pH 5.8 without Triton X-100. All these steps were executed at ambient temperature with continuous agitation on a rotary shaker. This treatment facilitated the replacement of SDS with Triton X-100 and enabled gel equilibration in a buffer suitable for enzymatic activity assessment. After equilibration, the gel was removed from the phosphate buffer, sealed in a container, and incubated at 37 °C for 18 h. It was then washed twice, each wash lasting 15 min, in 50 mL of 15 mM Tris-HCl buffer (pH 7.95). Following this, the gel was incubated in a staining solution under gentle agitation for a minimum of 5 h. Finally, it was kept in the dark until it was ready for imaging (Cèvallos et al., 1992; Costal-Oliveira et al., 2012). Just before use, the staining solution was prepared by mixing 5 mL of 0.1% Stains-All (Sigma-Aldrich, St. Louis, MO, United States) dissolved in pure formamide with an additional 5 mL of formamide, 20 mL of isopropanol, 1.5 mL of 1 M Tris-HCl buffer at pH 7.95, and water to reach a final volume of 100 mL (Green et al., 1973). Following the staining process, the gel was immersed in a destaining solution composed of 5% formamide, 20% isopropanol, and 15 mM Tris-HCl (pH 7.95) to enhance contrast. This treatment allowed enzymatic activity bands to appear distinctly against a deep blue background formed by undigested hyaluronic acid. The molecular weight of the active enzyme band was estimated using pre-stained protein molecular weight markers, which remained clearly visible against the dark-stained background of hyaluronan.

### 2.7 Protease activity

The proteolytic activity of these venom samples was assessed using fluorescein thiocarbamoyl–casein (Thermo Fisher Scientific, San Jose, California, United States) as a substrate (Twining, 1984; Huang et al., 1998). Each venom sample was thoroughly mixed with the substrate in individual 1.5-mL microtubes, which were sealed and incubated in a shaking water bath at 37 °C for 1 h. To terminate the enzymatic reaction, 120 μL of 5% trichloroacetic acid was added to each tube. The mixtures were then left to stand at room temperature for a minimum of 15 min to allow protein precipitation. Following incubation, the samples were centrifuged at 13,000 rpm for 3 min to pellet the insoluble proteins. From the resulting supernatant, a 30-μL aliquot was removed and diluted to a final volume of 2 mL using 500 mM Tris buffer at pH 8.5. Fluorescence measurements were conducted using a spectrofluorometer (RF-1500, Shimadzu), with excitation and emission wavelengths set at 490 nm and 525 nm, respectively. Caseinolytic activity was quantified by comparing the fluorescence intensity of each venom sample against that of trypsin, which served as a positive control. All measurements were performed in triplicate to ensure reproducibility.

### 2.8 Phospholipase A_2_ (PLA_2_) activity

Agarose (240 mg) were dissolved in 30 mL of phosphate-buffered saline (pH 8.1) in boiling water bath. The solution was cooled to 50 °C before adding 0.28 mL of the egg yolk (source of lecithin) diluted 1:4 with saline solution and 0.28 mL of 10 mM CaC1_2_ solution. This mixture (13 mL) was applied to plastic plates (90 × 80 mm), preheated to 50 °C, and allowed to gel. Subsequently, hollows with a diameter of 3 mm were excavated and filled with 20 μL of venom samples. The plates were kept at 37 °C for 18 h and stained with Stains-All dye to easily detect the diameter of the cleared egg yolk substrate zone. The control sample was prepared at a volume of 20 µL using PBS. The experiment was carried out in duplicate form (Gutiérrez et al., 1988; Costal-Oliveira et al., 2012; Li et al., 2013; Lee and Bae, 2016; Zhang et al., 2018).

### 2.9 Hemolytic activity

Sheep red blood cells (sRBCs) were isolated via centrifugation of sheep blood, washed three times with phosphate-buffered saline (PBS), which comprised 35 mM phosphate buffer and 150 mM sodium chloride at pH 7.2, and then pelleted through centrifugation at 2000 *g* for 5 min before being resuspended in PBS. A 50-µL portion of crude venom in PBS was combined with 50-µL of a suspension of sheep red blood cells at a concentration of 4% (v/v) in a micro-tube, then incubated for 1 h at 37 °C. Subsequently, the samples were centrifuged at 2000 *g* for 5 min, and then the resulting supernatants were poured into a 96-well plate where the release of hemoglobin was tracked by reading the absorbance at 450 nm with a microplate reader. PBS was used as a negative control, while 0.1% Triton X-100 was used as a positive control. The rate of hemolysis was determined by applying the following formula:

$$\text{Hemolysis rate }\left(\%\right)=\frac{A_{p}-A_{0}}{A_{T}-A_{0}}\times 100$$

where A_p_ denotes absorbance of the peptide sample, A_T_ denotes the absorbance of the positive control, and A_0_ represents the absorbance of the negative control.

### 2.10 Antibacterial activity

The antibacterial activity was evaluated by a growth inhibition assay conducted on a thin layer of agar. The bacteria *Escherichia coli* NBRC 3972 (a Gram-negative species), *S. aureus* NBRC 13276 (Gram-positive), and *Bacillus subtilis* NBRC 3009 (NITE Biological Resource Center, Chiba, Japan) were employed in this test. The bacteria were grown overnight at 37 °C in a Petri dish under aerobic conditions, followed by culture in liquid Luria Bertani broth consisting of 1% tryptone, 0.5% yeast extract, and 1% NaCl. A culture was incorporated into warm (∼50 °C) Luria Bertani broth supplemented with 2% agar at a concentration of 20 μL of bacterial culture per milliliter of medium. The resulting mixture was then poured into a Petri dish (with a diameter of 9 cm) to achieve a thin layer of agar approximately 1 mm in thickness. One or 2 μL of the venom solution (approximately 5 μg/μL) was applied to the agar surface. The plate was incubated at 37 °C for 12 h to observe the suppression of bacterial growth.

## 3 *In vivo* study

### 3.1 Animal models and experimental design

Rats that were male and albino (weighing 150–200 g) were acquired from the National Research Centre animal colony in Giza, Egypt. The experimental procedures were conducted in compliance with the ethical guidelines sanctioned by the Faculty of Science at Al-Azhar University, located in Assiut, Egypt. All animal protocols were approved by the Ethics Committee of Assiut University under approval number AZHAR 11/2025. The animals were acclimatized for 1 week in standard plastic cages under controlled conditions, with free access to food and water. Following acclimatization, the rats were randomly allocated into five groups (n = 10 per group).

- In the control group, 2 mL of buffer solution (pH 6.8) was administered orally.
- Group 2, which was used for disease control, was induced with nephrotoxicity using Taxol and received no additional treatment.
- Rats treated with Taxol were given *B. leptochelys* venom intraperitoneally at a dose of 10 μg/kg/day for 4 weeks.
- Rats treated with Taxol were administered intraperitoneally with *S. maurus palmatus* venom (10 μg/kg/day) for a period of 4 weeks.
- Rats treated with Taxol were injected intraperitoneally with 10 μg/kg/day of *L. quinquestriatus* venom for 4 weeks as part of Group 5.

### 3.2 Blood and tissue sampling

After a 4-week treatment period, animals were left without food overnight. Animals were deeply anesthetized for blood collection, with anesthesia being induced either by isoflurane delivery (2% in oxygen at a flow rate of 0.five to one L/min) or an intramuscular injection of 9.1 mg/kg sodium pentobarbital. Following confirmation of surgical anesthesia, blood samples were withdrawn via heparinized capillary glass tubes from the retro-orbital plexus. The blood that had been collected was allowed to clot, after which it was centrifuged at 3,000 rpm for 15 min at a temperature of 4 °C. The separated serum was split into portions and stored at −80 °C until biochemical analysis.

All animals were humanely killed by decapitation while still under deep anesthesia immediately following blood collection. The kidneys and hearts were then removed immediately following euthanasia. Hearts taken from chosen animals were first cleaned with saline, then dried and wrapped in aluminium foil before being stored at minus 80 °C for processing and examination via homogenization. Each kidney was divided into two portions. One portion was washed with saline, flash-frozen, and stored at −80 °C for biochemical analysis, while the other was fixed in 10% neutral buffered formalin for histopathological examination. Kidney tissue was homogenized in an ice-cold phosphate buffer solution with a concentration of 50 mM and pH 7.4 to create a 10% (w/v) homogenate. The homogenate was centrifuged at 7,000 rpm for 20 min, and the supernatant that formed was divided into portions and stored at −80 °C until it was needed.

### 3.3 Molecular and biochemical examinations

Serum biochemistry parameters, including urea, uric acid, creatinine, sodium, potassium, calcium, and albumin, were measured using DiaSys diagnostic kits from Germany on a Shimadzu UV-1201 spectrophotometer.

Renal homogenates at a concentration of 10% w/v were prepared in ice-cold 50 mM phosphate buffer (pH 7.4), then centrifuged at 5,000 rpm for 20 min. The supernatant obtained was utilized to evaluate oxidative stress parameters. Measurements were made of the activities of superoxide dismutase (SOD), glutathione peroxidase (GPX), catalase (CAT), and the concentrations of reduced glutathione (GSH), malondialdehyde (MDA), and nitric oxide (NO) using rat ELISA kits from SinoGeneClon Biotech Co. In China, with a Dynatech Microplate Reader.

The serum concentrations of tumor necrosis factor-alpha (TNF-α) and interleukin-1 beta (IL-1β) were measured using commercially available rat ELISA kits, specifically those from SinoGeneClon Biotech Co. In China, as per the manufacturer’s guidelines.

#### 3.3.1 Histopathological examination

Kidney tissues fixed with formalin were processed in accordance with a standard protocol that included dehydration, clearing in xylene, and embedding in paraffin wax. Sections of tissue (5 μm thick) were cut and stained with hematoxylin and eosin (H&E) for morphological assessment (Layton and Suvarna, 2013).

#### 3.3.2 Semi-quantitative histopathological scoring

For semi-quantitative assessment of renal injury, five randomly selected non-overlapping fields per kidney section (10 fields per rat, n = 5 rats per group) were examined under light microscopy (×400 magnification). Renal injury was scored by two independent blinded observers using a 0–4 grading system adapted from previous studies (Kim and Moon, 2012; Kudose et al., 2018). The following parameters were evaluated: tubular damage (0 = normal, 1 = mild affecting ≤10% of tubules, 2 = moderate affecting 11%–25%, 3 = severe affecting 26%–50%, 4 = extensive affecting >50%), interstitial hemorrhage (0 = absent, 1 = minimal, 2 = mild, 3 = moderate, 4 = severe), glomerular degeneration (0 = normal, 1 = mild, 2 = moderate, 3 = severe, 4 = complete degeneration), and inflammatory cell infiltration (0 = absent, 1 = minimal, 2 = mild, 3 = moderate, 4 = severe). A total pathological score (maximum 16) was calculated as the sum of individual parameter scores. Inter-observer agreement was assessed using Cohen’s kappa coefficient.

## 4 Results

### 4.1 Electrophoretic analysis of crude venoms

**FIGURE 1:**
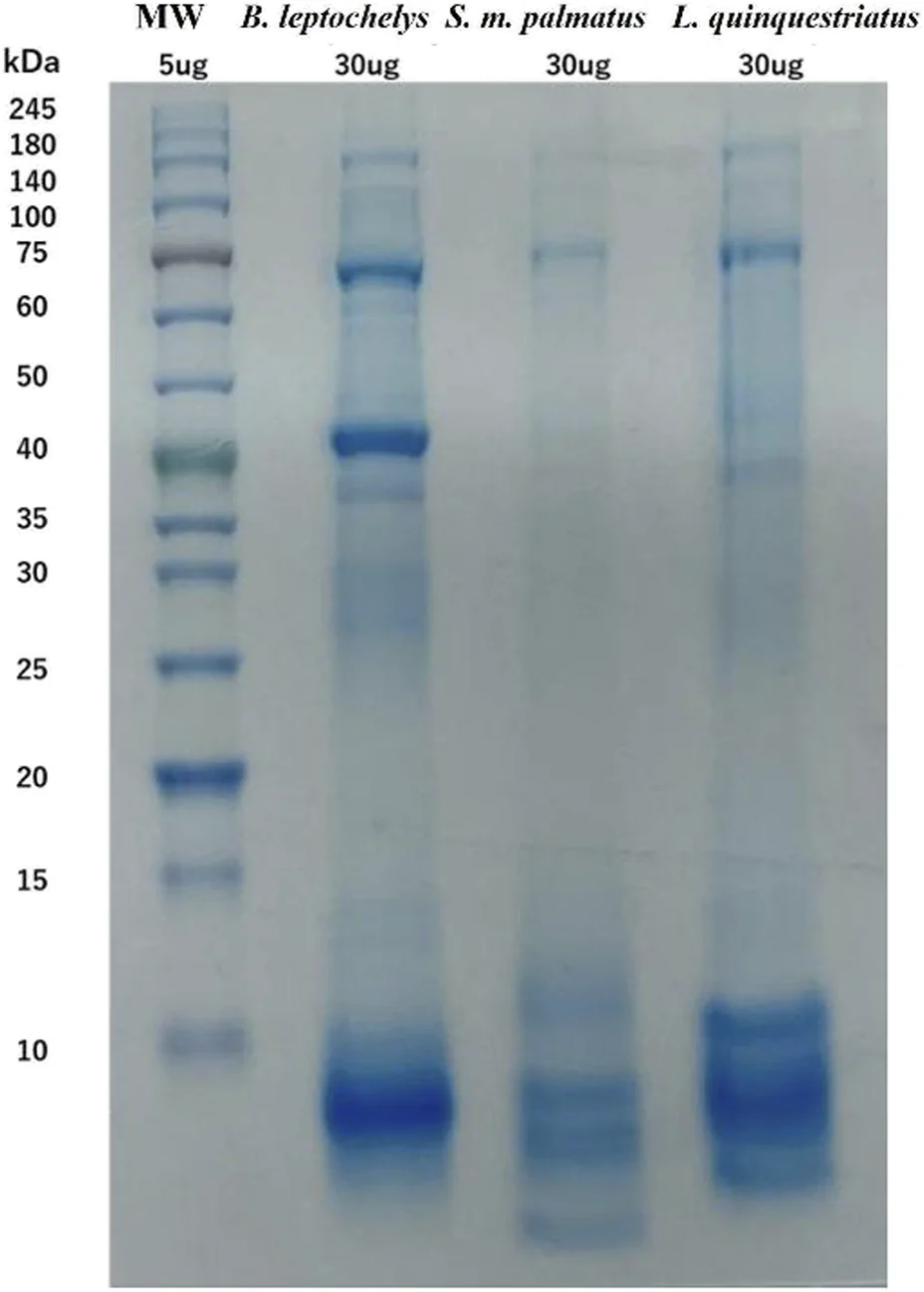
SDS-PAGE (15% separation gel) analysis of venoms of three scorpion species.

SDS-PAGE analysis of venom samples from *B. leptochelys*, *S. m. palmatus*, and *L. quinquestriatus* showed diverse protein profiles with molecular weights ranging from 4 to 130 kDa (Figure 1), and the protein composition and abundance differed significantly among the species. The venom of *B. leptochelys* showed the most diverse composition among the three, with (13) distinct bands. The SDS-PAGE gel showed a balanced spread of protein sizes, with notable strong bands at 89 kDa and 53 kDa, as well as a notably abundant high-intensity band at 6 kDa.

In contrast, the venom profile of *S. m. palmatus*, which contained (16) detectable bands, was characterized by a clear predominance of low molecular weight components. This region contained the most intense bands, featuring significant signals at 9 kDa and 7 kDa, along with a notably intense band at 6 kDa. Similarly, the venom of *L. quinquestriatus*, which exhibits (11) distinct bands, also had a high abundance of low molecular weight proteins. A prominent high-intensity band was visible at 95 kDa, with the most intense bands observed at 8 kDa and 7 kDa (Supplementary Table S1).

In all three species, protein bands were distributed across a wide range of molecular weights, including high, intermediate, and low molecular weight ranges. A consistent and notable band at 6–7 kDa was seen in all venoms, suggesting a conserved low molecular weight component, consisting of neurotoxic and antimicrobial peptides. The venoms of *S. m. palmatus* and *L. quinquestriatus* exhibited a similar pattern, marked by a high abundance of low molecular weight proteins, in contrast to *B. leptochelys*, which displayed a more balanced distribution with substantial high and low molecular weight components.

### 4.2 Mass spectrometric analysis

**FIGURE 2:**
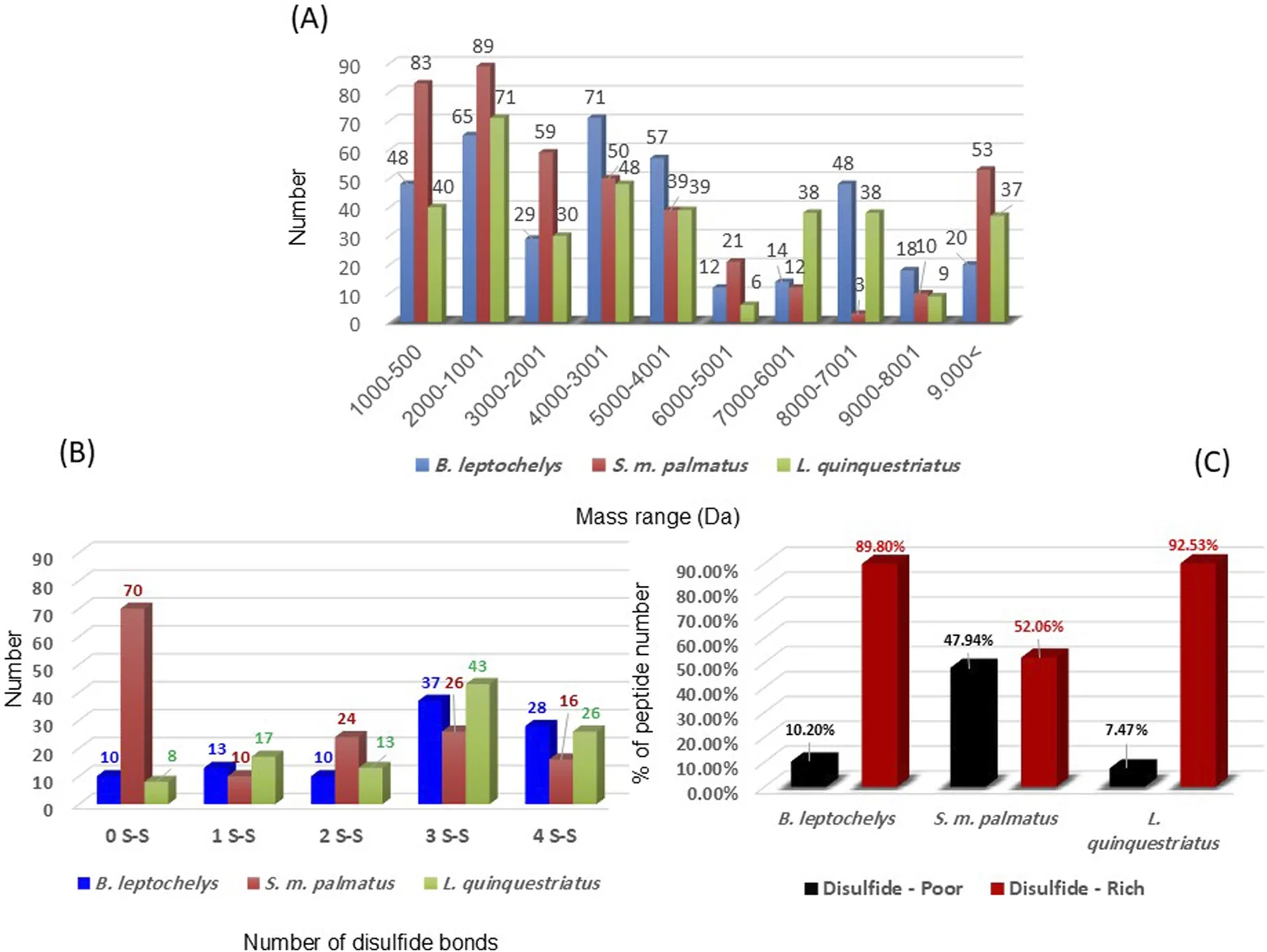
**(A)** LC/MS analysis of molecular mass distribution in *B. leptochelys*, *S. m. palmatus*, and *Leiurus quinquestriatus* venoms **(B)** Estimation and distribution pattern of compounds with disulfide bonds **(C)** Evaluation of disulfide-rich and -poor peptides in each species venom.

The peptide composition of crude venoms from *B. leptochelys*, *S. m. palmatus*, and *L. quinquestriatus* was analyzed using high-resolution LC/MS (Supplementary Figures S1–S3). The molecular masses of components were distributed within the range of 500–9,000 Da, as shown in Figure 2A.

In the venoms of *B. leptochelys*, *S. m. palmatus*, and *L. quinquestriatus*, a total of 382, 419, and 356 probable peptide components were identified (Supplementary Figures S2, S4, S6), respectively. Different distribution patterns were seen among the species. The venom of *B. leptochelys* exhibited a high number of components within the molecular weight ranges of 3,001–4,000 Da and 7,001–8,000 Da, which comprised 71 and 48 components, respectively. In contrast, the venom of *S. m. palmatus* contained a high number of components in the lower mass ranges of 500–1,000 Da and 1,001–2000 Da, with 83 and 89 components, respectively. *Leiurus quinquestriatus* venom, on the other hand, showed prominent peaks in the 1,001–2000 Da and 3,001–4,000 Da ranges, consisting of 71 and 48 components.

Venom constituents were treated with dithiothreitol to reduce disulfide-bonded peptides (DBPs) and then alkylated with iodoacetamide. The resulting mass shift, corresponding to an increase of 116.058 Da per disulfide bond, made it possible to identify the number of disulfide bonds in each DBP. A total of 98, 146, and 107 DBPs were found in the venoms of *B. leptochelys*, *S. m. palmatus*, and *L. quinquestriatus*, respectively (Supplementary Figures S3, S5, S7). A notable variation in disulfide bond frameworks was found among three species (Figure 7B). It is notable that a framework of six cysteines was found in most DBPs across the three scorpion species, forming three disulfide bridges (Figure 2B). The venom of *B. leptochelys*, *S. m. palmatus*, and *L. quinquestriatus* were found to contain NDBP,s accounting for 10.2%, 48.0%, and 7.5% of the total components, respectively (Figure 2C).

### 4.3 Hyaluronidase activity

**FIGURE 3:**
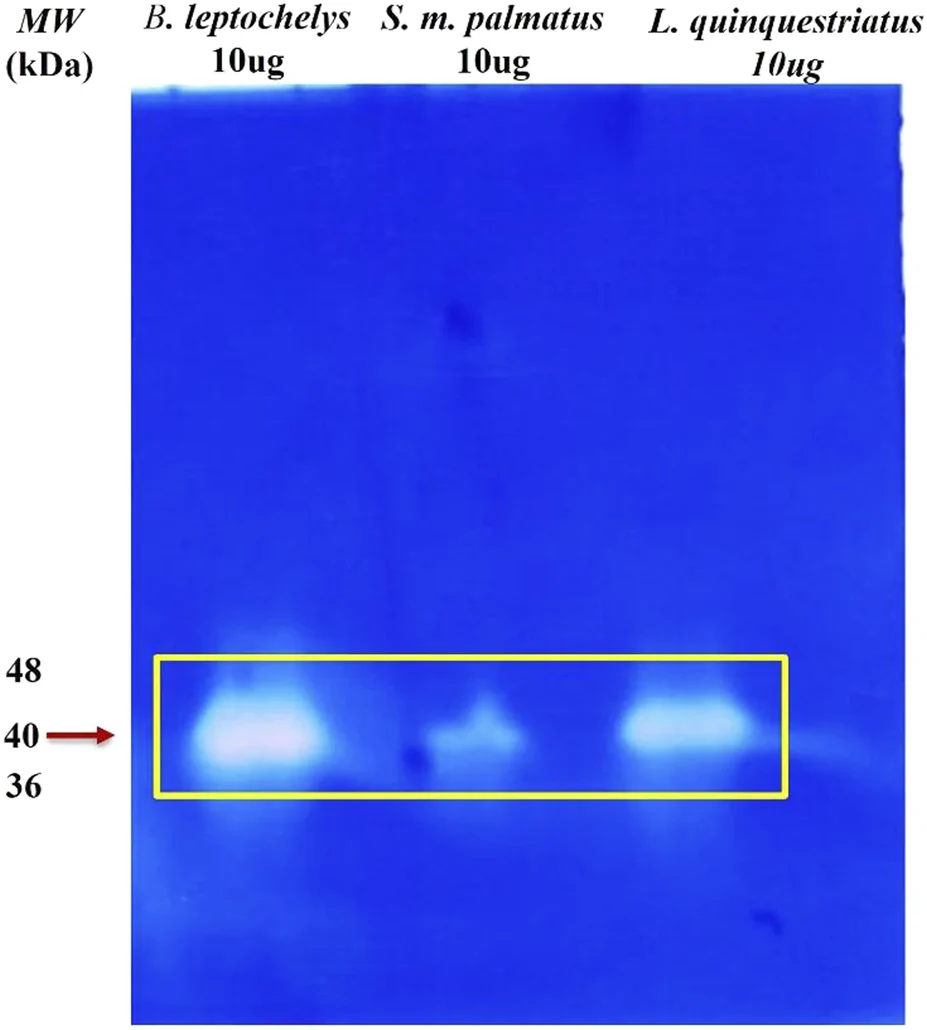
Hyaluronidase activity via zymogram analysis in the venom of *B. leptochelys, S. m. palmatus, and Leiurus quinquestriatus* species (The SDS–PAGE was copolymerized with hyaluronic acid).

Hyaluronidase is a vital venom component that is also referred to as a “spreading factor”. The process facilitates the release of toxins throughout the body by breaking down hyaluronic acid, a crucial component of the extracellular matrix. The degradation accelerates the distribution of other venom components throughout tissues, worsening local and systemic symptoms such as pain, inflammation, and significant swelling. The hyaluronidase activity in the venoms of three distinct scorpion species - *B. leptochelys, S. m. palmatus,* and *L. quinquestriatus* - was examined using a zymogram assay to assess the enzyme’s role in these species. Notably, hyaluronidase activity was found in all three species, with evidence of clear digestion zones at around 40 kDa as shown in Figure 3. The venoms of *B. leptochelys* and *L. quinquestriatus* (Buthidae family) showed significant enzymatic activity, suggesting a highly efficient system for venom dispersal. In contrast, *S. m. palmatus* (family Scorpionidae) venom exhibited weaker activity, due to lower enzyme abundance or structural differences like differential glycosylation. The presence of hyaluronidase in these scorpions, which are taxonomically distinct and belong to the Buthidae (*B. leptochelys and L. quinquestriatus*) and Scorpionidae (*S. m. palmatus*) families, implies that this enzyme plays a crucial role as a spreading factor in scorpion venom.

### 4.4 Proteolytic activity

**FIGURE 4:**
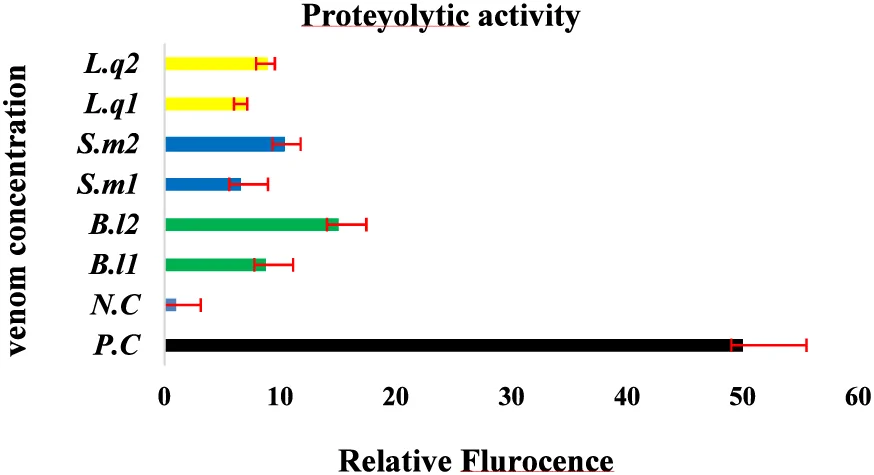
Protease essays using FTC-casein. The absorption value at 490 nm indicated that the proteolytic activity was present in (1) and (10) micrograms of crude venom. P.C: Positive control (trypsin, 1 µg); N.C: Negative control (distilled water) (B.l1, B.l2): *B. leptochelys* venom at 1 μg and 10 µg respectively (S.m1, S.m2): *S. m. palmatus* venom at 1 μg and 10 µg respectively; and (L.q1, L. q2): *Leiurus quinquestriatus* venom at 1 μg and 10 µg respectively.

Proteases are notable for their role in facilitating prey digestion and the spread of venom in scorpion stings. The pathological importance in the venoms of *B. leptochelys*, *L. quinquestriatus*, and *S. m. palmatus* remains unknown. To address this issue, we utilized a sensitive fluorescence-based assay to quantify proteolytic activity (Lima et al., 2025). The results showed minimal hydrolysis of FTC-casein, with activity levels at 490 nm much lower than those of the positive control (Figure 4). The results suggest that proteases are found in insignificant amounts or have low specific activity, which limits their contribution to the envenomation pathology of these species.

### 4.5 Phospholipase A_2_ activity

**FIGURE 5:**
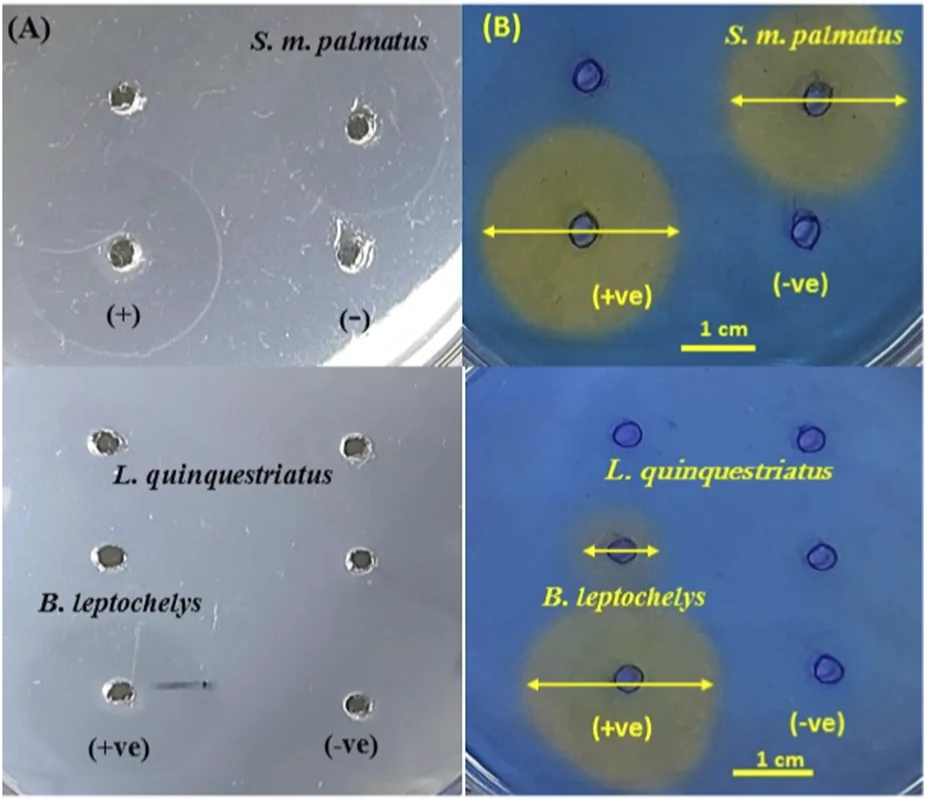
Phospholipase A2 (PLA2) activity of B. leptochelys, S. m. palmatus, and L. quinquestriatus scorpion species as determined by the indirect assay in agarose gel. Negative control (-ve): distilled water; Positive control (+ve): Bee Venom (*Apis mellifera*) and Scorpio maurus venom **(A,B)** represent agarose gel plates containing egg yolk as a lecithin source and CaCl2, without and with dye all stain, respectively.

Phospholipase A_2_ (PLA_2_) is a widespread venom component that breaks down membrane phospholipids, contributing to effects including hemolysis and inflammation. The presence and activity of scorpions can significantly differ across various families. This study evaluated PLA_2_ activity in the crude venoms of *B. leptochelys*, *L. quinquestriatus* (Buthidae), and *S. m. palmatus* (Scorpionidae) via an indirect agarose gel test using egg yolk as a lecithin source (Zhang et al., 2018; Megaly et al., 2025). The three species showed various levels of activity under standardized conditions (20 μg of venom). The venom of *S. m. palmatus* exhibited significant PLA_2_ activity, yielding a distinct halo with a diameter of 2.4 cm, like the positive control of 1 µg bee venom PLA_2_. In contrast, *B. leptochelys* venom exhibited merely a trace amount of activity, with a minimal halo diameter of 1.1 cm. No detectable halo formation was observed for *L. quinquestriatus* venom, which suggests that there was no measurable PLA_2_ activity at the tested dose (Figure 5, Table 1).

**TABLE 1:** Quantitative estimation of the PLA2 activity diameter of the crude venom’s doses, compared to the PLA2 activity diameter of bee venom as a positive control.

Scorpion species	Dose	Activity diameter
*B. leptochelys* *S. m. palmatus* *L. quinquestriatus*	20 μg 20 μg 20 μg	+- (1.1 cm) ++ (2.4 cm) -
PLA_2_ from bee venom	1 μg 0.1 μg 0.0625 μg	++ (2.4 cm) + (1.5 cm) +- (1.1 cm)

This inter-species variation demonstrates the differing venom methods used by family members with distinct characteristics. The potent activity in *S. m. palmatus* implies that PLA_2_ plays a role in the envenomation pathology, whereas its near or complete absence in *B. leptochelys* and *L. quinquestriatus* suggests that these species rely more heavily on other enzymes like hyaluronidase which is clearly exist in higher concentration in both *B. leptochelys* and *L. quinquestriatus* than *S. m. palmatus*, this is almost like internal equalization for the venom content. Also, these species may rely on other toxin families, including neurotoxins, for the efficacy of capturing prey.

### 4.6 Hemolytic activity

**FIGURE 6:**
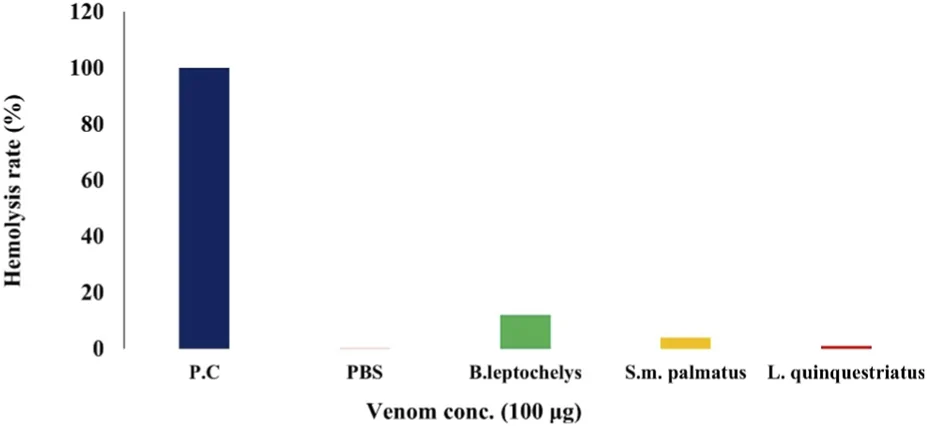
Effect of *B. leptochelys*, *S. m. palmatus*, and *Leiurus quinquestriatus* scorpion venom at high concentration (100 μg/mL) on hemolysis of Fresh sheep red blood cells (sRBCs). The amount of hemoglobin released from the erythrocyte suspension used to calculate the percentage of hemolysis, with measurements taken at a wavelength of 450 nm. A 1% (v/v) solution of Triton X-100 served as positive control (P.C), while PBS used as negative control.

Many scorpion venoms exhibit hemolytic activity, which is commonly caused by phospholipase A_2_ (PLA_2_) enzymes and cytolytic peptides. We assessed this activity in the venoms of *B. leptochelys*, *S. m. palmatus*, and *L. quinquestriatus* using a sheep red blood cell (sRBC) test (Gutiérrez et al., 1988; Costal-Oliveira et al., 2012; Luna-Ramirez et al., 2017). At a high concentration of 100 μg/mL, the venoms displayed distinctly different hemolytic characteristics (Figure 6). The venom of *S. m. palmatus*, which showed strong PLA_2_ activity, caused a modest hemolysis of around 3.9%. In contrast, the venom of *B. leptochelys*, which displayed only a trace amount of PLA_2_ activity, led to a significantly higher hemolytic value of approximately 12.0%. In contrast, the venom of *L. quinquestriatus*, which exhibited undetectable PLA_2_ activity, did not exhibit significant hemolysis (approximately 1.0%), like the PBS negative control. The significant hemolysis caused by *B. leptochelys* venom, despite its low PLA_2_ activity, implies the existence of other hemolytic components, like cytolytic peptides, in its venom. The substantial hemolysis by *B. leptochelys* venom, despite its weak PLA_2_ activity, suggests the presence of other hemolytic agents, such as cytolytic peptides, in its venom. The observed interspecific variation highlights differences in venom composition and predation tactics among the species under investigation.

### 4.7 Scorpion venoms reduce taxol-induced renal biochemical changes

To assess kidney damage and treatment methods, levels of substances in the blood were measured that show how well the kidneys are filtering waste and regulating electrolytes. Taxol® caused a substantial biochemical imbalance, increasing levels of nitrogenous wastes and potassium but decreasing calcium, sodium, and albumin. Consistently and significantly, subsequent therapy with scorpion venoms from *B. leptochelys, S. m. palmatus*, or *L. quinquestriatus* ameliorated this derangement, normalizing measured parameters toward baseline control levels with equivalent effectiveness across treatments (Table 2).

**TABLE 2:** Serum biochemical parameters in control, Taxol^®^, and venom-treated groups.

​	Control	Taxol®	Taxol®∼ B. leptochelys	Taxol®∼ S. m. palmatus	Taxol®∼ L. quinquestriatus
Urea (mg/dL)	±3.235.4	±5.1^*^85.4	±5.8^#^44.5	±3.1^#^39.2	±4.5^#^42.1
Creatinine (mg/dL)	±0.090.66	±0.21^*^1.9	±0.54^#^0.81	±0.54^#^0.69	±0.14^#^0.84
Uric acid (mg/dL)	±0.123.5	±0.21^*^6.8	±0.69^#^3.8	±0.19^#^3.6	±0.91^#^3.9
Calcium (mg/dL)	±0.649.9	7.2 ± 0.65^*^	±0.84^#^9.3	±0.23^#^9.6	±0.24^#^9.4
Sodium (mmol/L)	±6.8141.4	±5.2^*^112.1	±4.3^#^139.2	±7.1^#^140	±6.2^#^135.1
Potassium (mmol/L)	±0.664.5	±0.23^*^9.2	±0.94^#^4.9	±0.2^#^4.7	±0.74^#^5.1
Albumin (mg/dL)	4.6 ± 0.12	±0.41^*^3.2	±0.48^#^4.3	±0.51^#^4.4	±0.68^#^4.2

*Data are presented as mean ± SD (n = 10).

**Significantly different from the Control group (p ≤ 0.05).

# Significantly different from the Taxol® group (p ≤ 0.05).

Statistical analysis was performed using one-way ANOVA, followed by Duncan’s *post hoc* test.

### 4.8 Evaluation of renal oxidative stress

To assess the involvement of oxidative damage in Taxol-induced kidney damage, kidney tissue samples were analyzed for key indicator levels. Taxol® caused a state of significant pro-oxidation, marked by a considerable increase in malondialdehyde (MDA) and nitric oxide (NO), alongside a notable diminishment of antioxidant defenses, including reduced glutathione (GSH), superoxide dismutase (SOD), glutathione peroxidase (GPx), and catalase (CAT) activities (Table 3). Treatment with venoms from *B. leptochelys*, *S. m. palmatus*, or *L. quinquestriatus* reduced this oxidative imbalance. All three venom therapies efficiently reduced MDA and NO levels and returned GSH concentrations and antioxidant enzyme activities to levels comparable to those in the control group.

**TABLE 3:** Renal oxidative stress markers in control, Taxol^®^, and venom-treated groups.

​	Control	Taxol®	Taxol®∼ B. leptochelys	Taxol®∼ S. m. palmatus	Taxol®∼ L. quinquestriatus
MDA (pg/mL)	±6.149.4	±6.1^*^116.4	±9.1^#^56.5	±7.3^#^55.2	±12.1^#^59.5
NO (µmol/L)	±1.28.6	±2.8^*^26.8	±0.92^#^11.4	±1.0^#^9.8	±1.8^#^10.4
GSH (ng/mL)	±3.383.31	±5.4^*^66.5	±8.3^#^169.5	±14.1^#^177.4	±12.3^#^162.3
SOD (U/L)	±2.9158.3	±2.15^*^17.1	±4.4^#^44.5	±6.66^#^52.4	±3.4^#^48.5
GPx (U/L)	±19.2226.4	±8.1^*^67.3	±14.9^#^195	±13.2^#^219.5	±11.9^#^199
CAT (U/L)	±2.635.5	±2.4^*^15.4	±1.3^#^28.8	±4.1^#^32.1	±3.3^#^29.5

* Data are presented as mean ± SD (n = 10). *.

*Significantly different from the Control group (p ≤ 0.05).

# Significantly different from the Taxol® group (p ≤ 0.05).

Statistical analysis was performed using one-way ANOVA, followed by Duncan’s *post hoc* test.

### 4.9 Assessment of systemic inflammation

Taxol-induced nephropathy’s associated inflammatory response was evaluated by quantifying serum levels of pro-inflammatory cytokines. A significant systemic inflammatory response was triggered by Taxol intoxication, characterized by marked increases in serum levels of tumors necrosis factor-alpha (TNF-α) and interleukin-1 beta (IL-1β) compared to the control group (Supplementary Figures S5A,B). The administration of venom from *B. leptochelys*, *S. m. palmatus*, or *L. quinquestriatus* effectively reduced this inflammatory cascade. Significant treatments for venom exposure decreased the levels of both TNF-alpha and IL-1 beta, returning them to concentrations comparable to those found in the healthy control group.

#### 4.9.1 Histopathological examination

**FIGURE 7:**
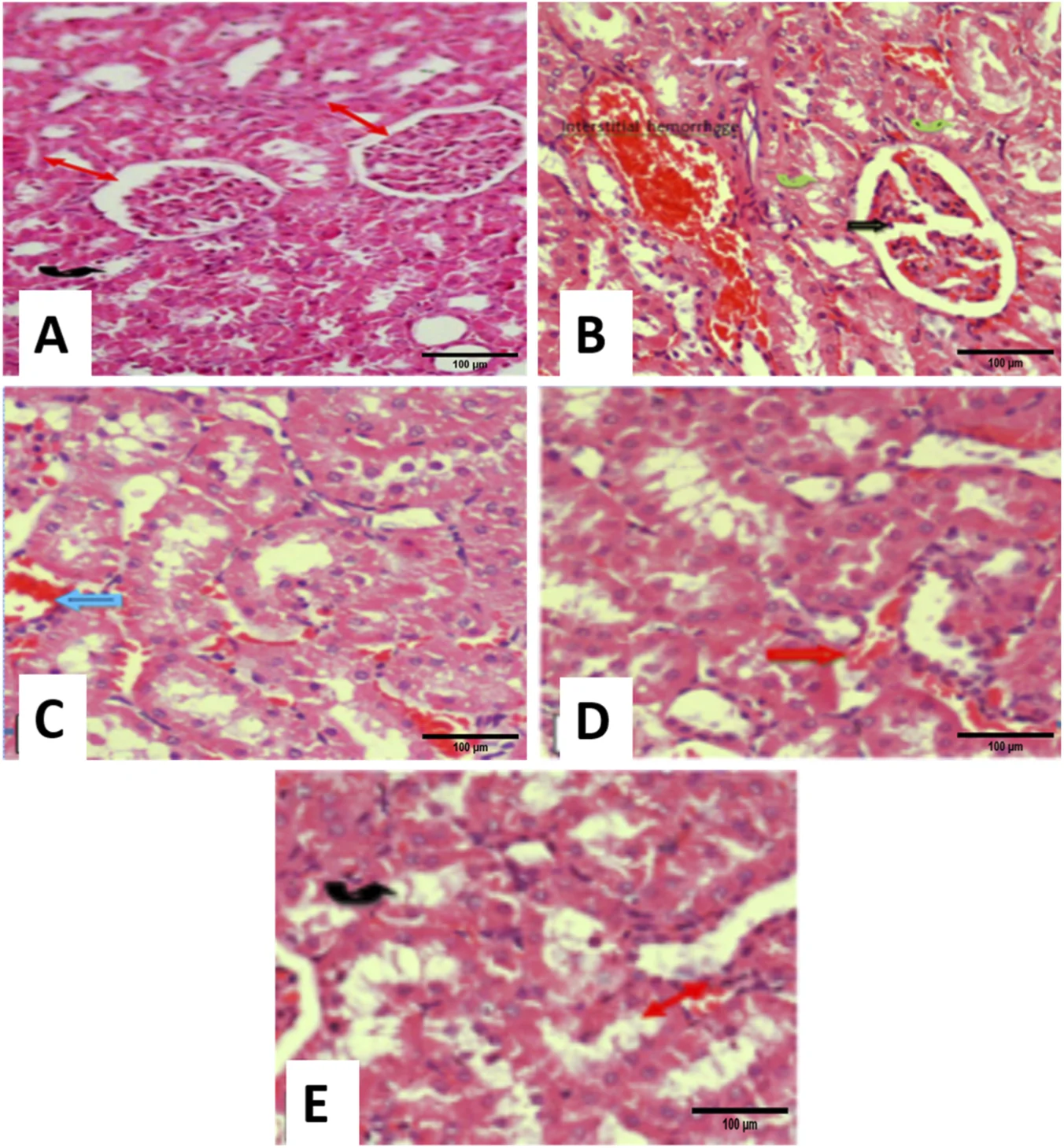
Representative photomicrographs of kidney sections (H&E stain, 400×) showing the histopathological changes in each experimental group. Semi-quantitative scoring of renal injury (tubular damage, interstitial hemorrhage, glomerular degeneration, and inflammatory cell infiltration) **(A)** Control rat showing normal glomeruli (red arrow) and tubules (curved black arrow) **(B)** Taxol®-treated rat showing severe glomerular degeneration (black arrow), wide urinary space, vacuolar degeneration (green arrow), and interstitial hemorrhage **(C)** Taxol® + *Leiurus quinquestriatus* treated group revealed improved architecture with minimal interstitial hemorrhage (blue arrow) **(D)** Taxol® + *B. leptochelys* treated group showing preserved glomeruli with mild interstitial hemorrhage (red arrow) **(E)** Taxol® + *S. m. palmatus* treated group revealing congested blood vessel (red arrow) and preserved renal tubules (curved black arrow). Scale bar = 50 μm.

The morphology of renal tissue was studied using light microscopy in order to link biochemical data with structural damage. The kidneys from the control rats showed a normal histoarchitecture of the glomeruli and renal tubules (Figure 7A). In contrast, rats treated with Taxol alone exhibited severe pathological changes, including glomerular degeneration with an expansion of the urinary space, interstitial hemorrhage, fibrosis, and damage to the tubular epithelial cells characterised by pyknosis, karyolysis, and vacuolar degeneration (Figures 7B,C). Treatment with any of the three scorpion venoms in combination with Taxol conferred notable histological protection. The kidneys from these groups maintained the normal structure of glomeruli and tubules, with minimal evidence of interstitial hemorrhage seen in some samples, showing a significant reduction of Taxol-induced nephrotoxic damage.

#### 4.9.2 Semi-quantitative histopathological scoring

To quantitatively validate the visual observations from H&E staining, we performed semi-quantitative scoring of renal injury (Table 4). The control group showed minimal pathological changes (total score 0.4 ± 0.2). In contrast, Taxol® administration induced severe renal injury, characterized by extensive tubular damage (3.8 ± 0.2), moderate-to-severe interstitial hemorrhage (3.2 ± 0.3), severe glomerular degeneration (2.9 ± 0.3), and mild-to-moderate inflammatory cell infiltration (1.6 ± 0.2), resulting in a total score of 11.5 ± 1.2 (p ≤ 0.05 vs. control). Treatment with *B. leptochelys*, *S. m. palmatus*, or *L. quinquestriatus* venoms significantly reduced all pathological parameters. The most pronounced protection was observed in the *L. quinquestriatus* group (total score 3.2 ± 0.5), followed by *B. leptochelys* (3.7 ± 0.8) and *S. m. palmatus* (4.5 ± 0.7). These quantitative data confirm the qualitative observations from Figure 7 and demonstrate that scorpion venom treatment effectively preserves renal architecture against Taxol®-induced injury, consistent with the biochemical markers of renal function and oxidative stress.

**TABLE 4:** Semi-quantitative histopathological scoring of renal injury.

Histological parameter	Score criteria	Control	Taxol®	Taxol®∼ B. leptochelys	Taxol®∼ S. m. palmatus	Taxol®∼ L. quinquestriatus
Tubular damage (0–4)	0 = normal; 1 = mild (≤10%); 2 = moderate (11%–25%); 3 = severe (26%–50%); 4 = extensive (>50%)	0.2 ± 0.1	3.8 ± 0.2*	1.1 ± 0.3^#^	1.3 ± 0.2^#^	0.9 ± 0.2^#^
Interstitial hemorrhage (0–4)	0 = absent; 1 = minimal; 2 = mild; 3 = moderate; 4 = severe	0.0 ± 0.0	3.2 ± 0.3*	1.2 ± 0.3^#^	1.5 ± 0.3^#^	1.1 ± 0.2^#^
Glomerular degeneration (0–4)	0 = normal; 1 = mild; 2 = moderate; 3 = severe; 4 = complete degeneration	0.1 ± 0.1	2.9 ± 0.3*	0.9 ± 0.2^#^	1.1 ± 0.2^#^	0.8 ± 0.1^#^
Inflammatory cell infiltration (0–4)	0 = absent; 1 = minimal; 2 = mild; 3 = moderate; 4 = severe	0.1 ± 0.1	1.6 ± 0.2*	0.5 ± 0.2^#^	0.6 ± 0.2^#^	0.4 ± 0.1^#^
Total score (0–16)	Sum of above	0.4 ± 0.2	11.5 ± 1.2*	3.7 ± 0.8^#^	4.5 ± 0.7^#^	3.2 ± 0.5^#^

* Data are presented as mean ± SD (n = 10). *.

*Significantly different from the Control group (p ≤ 0.05).

# Significantly different from the Taxol® group (p ≤ 0.05).

Statistical analysis was performed using one-way ANOVA, followed by Duncan’s *post hoc* test.

## 5 Discussion

Bioactive compounds found in animal venoms, especially those from scorpions, constitute a substantial source of potential pharmaceuticals (Johana Vargas Munoz and Estrada-Gomez, 2014; Ortiz et al., 2015; Bordon et al., 2020). This study offers a comparative enzymatic, mass profiling and biochemical analysis of the venoms from three Egyptian scorpion species, *B. leptochelys*, *S. m. palmatus*, and *L. quinquestriatus* and assesses their therapeutic potential against Taxol®-induced nephrotoxicity.

Initial SDS-PAGE analyses uncovered distinct venom compositions specific to each species, in accordance with prior findings of significant interspecific diversity in scorpion venoms (Keskin and Koç, 2006; Borges et al., 2010; de Roodt et al., 2010; Khoobdel et al., 2013; Xu et al., 2014; Díaz-García α et al., 2015). Our SDS-PAGE profiles indicated that low molecular weight proteins (4–9 kDa) were predominantly present, which is consistent with the previously documented abundance of neurotoxins and antimicrobial peptides within this size range (Possani et al., 2000; Escobar et al., 2003; Velásquez and Escobar, 2004; Ding et al., 2014). We also noted notable species-specific patterns, with *B. leptochelys* having a more balanced distribution across molecular weights, and *S. m. palmatus* and *L. quinquestriatus* venoms showing enrichment in lower mass components.

Research has shown that enzymes with high molecular weights, like hyaluronidases, are found universally in scorpion venoms (Pessini et al., 2001; Morey et al., 2006; Batista et al., 2007; Seyedian et al., 2010). These enzymes act as spreading factors, facilitating the spread of venom throughout the prey’s tissues by degrading the glycosaminoglycans in the connective tissue, thus enabling systemic envenomation (Morey et al., 2006). Notably, the hyaluronidase activity in scorpion venoms is significantly higher than in snake venoms (de Souza et al., 2015; Guerra-Duarte et al., 2019) and spider venoms. The activity of hyaluronidase has been found in the venoms of diverse scorpion species, with molecular weights of the relevant enzymes ranging from 40 to 60 kDa (Pessini et al., 2001; Morey et al., 2006; Batista et al., 2007; Costal-Oliveira et al., 2012; Rodríguez-Ravelo et al., 2013; Díaz-García α et al., 2015; de Oliveira Domingos et al., 2018; Megaly et al., 2025) Rapid venom dissemination and the quick onset of symptoms in patients with scorpion bites are significant obstacles to effective patient treatment, primarily due to high hyaluronidase activity in scorpion venoms, which can cause both issues (Ahmadi et al., 2020). Our detection of hyaluronidase activity in all three species confirms its widespread role as a crucial “spreading factor” in scorpion venoms (Pessini et al., 2001; Morey et al., 2006; Batista et al., 2007; Seyedian et al., 2010). Activity observed in *B. leptochelys* and *L. quinquestriatus*, especially, is consistent with research indicating higher levels of hyaluronidase in scorpion venom than in other venoms, and its correlation with rapid symptom development and systemic envenomation (de Souza et al., 2015; Guerra-Duarte et al., 2019; Ahmadi et al., 2020). The enzymatic activity likely facilitates the spread of neurotoxins targeting sodium and potassium channels, which contributes to the severe systemic symptoms associated with envenomation by Buthidae species (Mendes et al., 2023; He et al., 2025). Megaly (2025) recently expanded this catalog by confirming hyaluronidase activity in both whole and soluble venoms of *Apis amoreuxi*, *Apis australis*, or *Apis bicolor* using zymogram assays. Consistent with these findings, our study detected hyaluronidase activity in all three Egyptian scorpion species examined (Figure 2). The presence of this enzyme in these Egyptian species expands the geographical and taxonomic records of this functionally vital venom component.

In contrast, the proteolytic activity across the three venoms was consistently low (Figure 4). Several studies support this finding by showing that scorpion venoms typically have low protease activity relative to other venomous animals, such as snakes (Almeida et al., 2002; Seyedian et al., 2010; Costal-Oliveira et al., 2012; Brazón et al., 2014; Ortiz et al., 2015). Some scorpion venom proteases have been linked to tissue damage, bleeding, and the activation of toxin precursors (Magalhães, 1946; Renner et al., 1983; Almeida et al., 2002), yet their low activity here implies that tissue degradation is not a main cause of disease in these species. Their role may be more supplementary, potentially involving the regulation of venom components or mildly elevating local tissue permeability.

A notable observation was the significant interspecific variation in phospholipase A_2_ (PLA_2_) activity. The venom from *S. m. palmatus* displayed significant PLA_2_ activity like that of the positive control, whereas *B. leptochelys* exhibited only minimal PLA_2_ activity and *L. quinquestriatus* none. The pattern observed here is in direct contrast to the reported absence of PLA_2_ in certain Androctonus species (Almeida et al., 2002; Valdez-Cruz et al., 2004; 2007; Venancio et al., 2013; Díaz-García α et al., 2015) yet it aligns with the documented presence of a hemolytic PLA_2_ in *Scorpio maurus palmatus* (Louati et al., 2013). The potent hemolytic activity seen in *B. leptochelys* venom, despite its low PLA_2_ activity, implies the presence of alternative cytolytic agents, like antimicrobial or cytolytic peptides. This shows a different approach to venom, where *S. m. palmatus* uses PLA_2_ to disrupt membranes, whereas the other two species might rely more on neurotoxins and other peptide groups to subdue their prey.

The antibacterial assays demonstrated restricted, species-specific antibacterial activity (see Table 2). The modest effects seen in *Escherichia coli* and *B. subtilis*, as well as the absence of activity in *S. aureus*, align with the mixed antimicrobial profiles observed in other scorpion venoms (Montaño-Pérez and Vargas-Albores, 2002; Carmo et al., 2014; Harrison et al., 2014; Park et al., 2020; Rádis-Baptista and Konno, 2020; Seyfi et al., 2020). The lack of activity in hydrophobic fractions (40–55 min) for these species implies that their antimicrobial peptides, if they exist, may be linear, and elute before those hypothesized to contain disulfide bonds for other species. Our mass spectrometry data further highlights the diversity inherent in its composition.

Mass spectrometry analysis resolved hundreds of peptide constituents per venom, with molecular mass distributions varying significantly among the three species. The comparative mass spectrometric profiling revealed marked interspecific variation in the relative abundance of NDBPs *versus* DBPs, which correlates closely with the distinct enzymatic profiles observed. Specifically, the high proportion of disulfide-bonded peptides in *B. leptochelys* and *L. quinquestriatus* (89.8% and 92.5%, respectively) classifies their venoms as “disulfide-rich,” typical of neurotoxin-dominated Buthidae venoms (Pimenta et al., 2001; Batista et al., 2004; Caliskan et al., 2006; Favreau et al., 2006; Yoshimoto et al., 2019). This structural architecture, featuring conserved six-cysteine frameworks forming three disulfide bridges, produces compact, conformationally constrained peptides that are highly stable and target ion channels with high specificity (Possani et al., 1999; Quintero-Hernández et al., 2013). The predominance of DBPs aligns with the potent neurotoxic strategy employed by Buthidae species, which rely heavily on sodium and potassium channel-modulating toxins for rapid prey immobilization. Importantly, the high hyaluronidase activity observed in both Buthidae species may be functionally linked to this DBP-rich composition: the structural stability conferred by disulfide-rich architectures may preserve enzyme-active conformations and facilitate sustained enzymatic activity, thereby enhancing venom dissemination through connective tissues (de Souza et al., 2015; Guerra-Duarte et al., 2019). Conversely, *L. quinquestriatus* exhibited no detectable PLA_2_ activity. We propose that this absence is compensated by two complementary mechanisms: first, exceptionally high hyaluronidase activity (comparable to *B. leptochelys*), which promotes rapid systemic distribution of neurotoxins; and second, an abundance of potent neurotoxic DBPs that directly incapacitate prey via ion channel blockade, rendering membrane-disruptive PLA_2_ enzymes unnecessary for effective prey capture. This represents a specialized venom strategy prioritizing neurotoxicity and diffusion over local cytolytic damage (Santibáñez-López et al., 2022; Štundlová et al., 2022). In striking contrast, *S. m. palmatus* venom contained the highest proportion of NDBPs (48.0%) and an enrichment of low-molecular-weight peptides (500–2000 Da), which may include the enzymatic and cytolytic components responsible for its unique bioactivity profile. NDBPs are typically linear, flexible, and amphipathic properties that facilitate membrane interaction and disruption (Almaaytah and Albalas, 2014). This compositional profile directly corresponds with the high PLA_2_ activity observed exclusively in this species (halo diameter 2.4 cm, comparable to bee venom control). The membrane-active NDBPs likely act synergistically with PLA_2_ enzymes, promoting substrate accessibility and enhancing phospholipid hydrolysis. The relatively lower hyaluronidase activity in *S. m. palmatus* suggests an alternative venom strategy that prioritizes potent local cytotoxic and phospholipolytic effects over extensive tissue diffusion consistent with the evolutionary position of Scorpionidae as a more primitive lineage relying on mechanical force (large pedipalps) combined with cytotoxic venom rather than rapid neurotoxin (Abdel-Rahman et al., 2009; Howard et al., 2019). Of note, the presence of peptides in the 3–8 kDa match cross all species matches the mass range of long-chain neurotoxins and other bioactive peptides (De Lima et al., 2007; Gurevitz et al., 2007; Yoshimoto et al., 2019).

When these venom, strategies are compared, a striking pattern of biochemical trade-off emerges. Buthidae species have apparently invested in hyaluronidase to maximize neurotoxin spread while minimizing or eliminating PLA_2_, whereas *S. m. palmatus* has retained PLA_2_ as a primary weapon while exhibiting lower hyaluronidase activity (Huang et al., 2026). This biochemical equalization where different enzymatic compositions achieve functionally comparable venom potency suggests that diverse molecular solutions can evolve to meet similar ecological demands. Future quantitative proteomic studies comparing enzyme abundance and specific activity will be valuable to test this hypothesis directly.

Taken together, these findings suggest that variations in molecular mass distribution and disulfide-bond organization are closely associated with venom functional specialization among the three scorpion species. Venoms enriched in structurally stable disulfide-bonded peptides appear to correlate with elevated hyaluronidase activity, potentially enhancing venom dissemination through tissues, whereas venoms containing higher proportions of low-molecular-weight NDBPs are more strongly associated with increased PLA_2_ activity and membrane-disruptive potential. These differences likely reflect distinct ecological and evolutionary adaptations influencing prey immobilization, defense mechanisms, and venom delivery strategies.

The most promising outcome of this research was the substantial kidney-protecting effect of all three venoms against Taxol-induced kidney damage. Administration of Taxol® resulted in a characteristic pattern of kidney damage: reduced filtering ability (increased levels of creatinine, urea, and uric acid), disturbances in electrolyte balance, oxidative stress (elevated MDA and NO, low levels of GSH, SOD, GPx, and CAT), inflammation (elevated TNF-α and IL-1β), and significant cell damage under the microscope (Baş and Nazıroğlu, 2019; Beheshtizadeh et al., 2024; El-Qassas et al., 2025). Treatment with each scorpion venom effectively reversed these alterations, which in turn normalized biochemical parameters, restored antioxidant defenses, suppressed pro-inflammatory cytokines, and markedly preserved renal histoarchitecture.

Scorpion venoms are rich in disulfide-bridged peptides that target voltage-gated ion channels, including K^+^, Na^+^, Ca^2+^, and Cl^−^ channels. Among these, K^+^ channel blockers are particularly relevant to NLRP3 inflammasome regulation because K^+^ efflux from the cytoplasm is considered a central upstream signal required for NLRP3 activation (Muñoz-Planillo et al., 2013). When K^+^ channel-blocking peptides inhibit potassium efflux, this suppresses inflammasome assembly, caspase-1 activation, and subsequent maturation and release of IL-1β. Consequently, the reduction in IL-1β also attenuates downstream inflammatory mediators, including TNF-α. Notably, all three scorpion species examined in this study are known to contain K^+^ channel-blocking peptides: *S*. *m*. *palmatus* contains maurotoxin, a four-disulfide bridge toxin that potently blocks various K^+^ channel subtypes (Kharrat et al., 1997); *B. leptochelys* contains BlTx1 and BlTx2, which show distinct functional selectivity for Kv4.1 channels (Levy et al., 2026); and *L*. *quinquestriatus* contains agitoxin 1, 2, and 3, which are pore-blocking peptides targeting voltage-gated potassium channels (Garcia et al., 1994). We therefore propose that the nephroprotective effects observed in our study may be partly attributed to these K^+^ channel-blocking peptides, which could suppress NLRP3 inflammasome activation and subsequent IL-1β/TNF-α release, thereby attenuating the inflammatory cascade in Taxol®-induced kidney injury. We acknowledge that this mechanistic link remains speculative based on our current data, and we recommend that future studies involving fractionation-guided bioassays and selective K^+^ channel blockers be conducted to definitively establish this pathway.

Beyond this specific K^+^ channel/NLRP3 mechanism, the venoms also exert broader protective effects that contribute to their overall nephroprotective action. The nephroprotective effects of these compounds can be attributed to the potent antioxidant and anti-inflammatory properties of the venom components. The venoms mitigated oxidative stress, a primary cause of Taxol-induced cell death, by scavenging reactive oxygen species and enhancing endogenous antioxidant enzymes (Lopez-Novoa et al., 2011; Flora et al., 2012). The decrease in pro-inflammatory cytokines (TNF-α, IL-1β) at the same time probably limited the inflammatory cascade that worsened the damage to the renal tissue (Karakoc et al., 2025). The comparable effectiveness of the three venoms, despite their differing compositions, implies that multiple peptide families within scorpion venoms, whether neurotoxins, antimicrobial peptides, or other unknown components, may possess these protective bioactive properties.

While the preceding discussion has established the biochemical diversity, enzymatic trade-offs, and notable nephroprotective efficacy of the three Egyptian scorpion venoms in a preclinical model, translating these promising findings into clinical practice requires careful consideration of several practical and regulatory challenges. Key concerns include the potential immunogenicity of venom proteins upon repeated administration, which may be mitigated through peptide fractionation, PEGylation, or synthetic analogs; batch-to-batch variability due to biological factors, resolvable via recombinant production or chemical synthesis; and the need to transition from intraperitoneal administration in preclinical studies to clinically feasible routes such as subcutaneous injection or targeted nanoparticle delivery. The sub-lethal dose used (10 μg/kg/day) offers a favorable risk-benefit profile, being well below reported LD_50_ values. Regulatory precedent exists, as chlorotoxin (TM-601) from *L. quinquestriatus* has completed Phase I/II trials for glioma, supporting GMP-compliant development pathways. For chemotherapy patients, the benefit of renal preservation may outweigh risks with careful patient selection and monitoring.

To overcome these hurdles, we recommend a stepwise development pipeline: (i) fractionation-guided identification of the specific peptides responsible for nephroprotection; (ii) chemical synthesis or recombinant production of lead peptides; (iii) *in vivo* pharmacokinetic and toxicokinetic studies; (iv) formulation development for targeted delivery; and (v) Phase I clinical trials in chemotherapy patients to establish safety, tolerability, and preliminary efficacy.

## 6 Conclusion

This investigation clarifies the intricate and species-specific venom compositions of three Egyptian scorpions, associating their unique enzymatic functions (hyaluronidase, PLA_2_) with possible ecological and pathological implications. A particularly striking finding is the biochemical trade-off observed between the two Buthidae species (*L. quinquestriatus* and *B. leptochelys*) and the Scorpionidae species (*S. m. palmatus*): while *L. quinquestriatus* lacks PLA_2_ activity entirely, it exhibits high hyaluronidase efficiency, whereas *S. m. palmatus* displays the opposite pattern with high PLA_2_ activity and lower hyaluronidase activity. This inverse relationship, together with the marked variation in DBP/NDBP ratios (48% NDBPs in *S. m. palmatus versus* only 7.5% in *L. quinquestriatus*), reflects distinct evolutionary strategies - Buthidae species prioritizing neurotoxin dissemination via hyaluronidase, and Scorpionidae relying on PLA_2_-mediated cytotoxicity supplemented by mechanical force from large pedipalps. Such functional venom evolution demonstrates how diverse molecular solutions can arise under different selective pressures to achieve comparable predatory success. The study also reveals a previously unnoted therapeutic capability: the ability of these venoms to alleviate drug-induced kidney damage through highly effective antioxidant and anti-inflammatory processes. These findings not only contribute to the comparative toxicology of scorpion venoms but also make them a promising source of novel bioactive compounds for developing additional therapies to counteract the dose-limiting renal toxicity of essential chemotherapeutic agents such as Taxol. Future research should concentrate on identifying and detailing the venom peptides accountable for these noted reno-protective effects. While our study provides comprehensive enzymatic and mass profiling, future bottom-up proteomics studies are needed to definitively identify and quantify the specific enzymes (PLA_2_, hyaluronidase, proteases) responsible for the observed activities. From a translational perspective, key hurdles including immunogenicity, batch-to-batch variability, and regulatory standardization must be addressed; however, the successful clinical development of chlorotoxin (TM-601) from *L. quinquestriatus* venom for glioma therapy provides a precedent for the feasibility of venom-derived pharmaceuticals. We further recommend fractionation-guided bioassays, *de novo* sequencing, chemical synthesis, and rational design to optimize lead peptides for potential therapeutic applications against drug-induced nephrotoxicity.

## Data Availability

The datasets presented in this article are not readily available because the specific LC-MS data measuring only the molecular masses of intact peptides obtained in this study are not accepted by standard public proteomics repositories (such as ProteomeXchange), which are strictly limited to LC-MS/MS data with protein identification results. Requests to access the datasets should be directed to the corresponding author, Mohammed Abdel-Wahab, at mnassar919@azhar.edu.eg.
