# Supplementary material for: Pharmacological characterization and nephroprotective potential of venoms from three egyptian scorpion species
Source: Front Pharmacol. 2026 Aug 28;17:1795320. doi: 10.3389/fphar.2026.1795320 (PMC13561842; doi:10.3389/fphar.2026.1795320)
Supplement: Supplementary file 1 [file Supplementaryfile1.docx]

**Table (S1).** Scorpion venoms electrophoretic profiles (15% separation gel) with CBB G-250 staining.

| **Protein band**  **(kDa)** | ***B. leptochelys*** | ***S. m. palmatus*** | ***L. quinquestriatus*** |
| --- | --- | --- | --- |
| **130** |  |  | **+** |
| **127** | **+** | **+** |  |
| **116** |  | **+** | **+** |
| **112** | **+** |  |  |
| **95** |  | **+** | **+** |
| **89** | **+** |  |  |
| **84** |  |  | **+** |
| **80** | **+** |  |  |
| **74** | **+** |  |  |
| **60** | **+** |  |  |
| **56** |  |  | **+** |
| **53** | **+** | **+** |  |
| **48** |  | **+** | **+** |
| **45** | **+** |  |  |
| **38** |  | **+** |  |
| **35** | **+** | **+** |  |
| **31** |  | **+** |  |
| **30** | **+** |  |  |
| **29** |  | **+** |  |
| **27** |  | **+** |  |
| **21** |  | **+** |  |
| **14** |  | **+** | **+** |
| **12** | **+** |  | **+** |
| **11** | **+** |  |  |
| **9** |  | **+** |  |
| **8** |  |  | **+** |
| **7** |  | **+** | **+** |
| **6** | **+** | **+** |  |
| **5** |  |  | **+** |
| **4** |  | **+** |  |

**Table (S2)**: Monoisotopic molecular masses (MM) of components in the ***B. leptochelys*** venom detected by LC/MS analysis.

| **MM** | **MM** | **MM** | **MM** | **MM** |
| --- | --- | --- | --- | --- |
| 529.31 | 967.595 | 1665.80 | 2723.45 | 3416.63 |
| 544.32 | 997.59 | 1668.71 | 2812.22 | 3422.57 |
| 553.39 | 1013.55 | 1672.01 | 2906.32 | 3422.58 |
| 561.26 | 1018.5 | 1718.99 | 2957.50 | 3471.54 |
| 569.29 | 1035.56 | 1734.89 | 2980.15 | 3474.49 |
| 578.80 | 1046.43 | 1774.67 | 2980.16 | 3488.39 |
| 588.36 | 1052.67 | 1778.94 | 3001.26 | 3526.43 |
| 592.15 | 1075.60 | 1788.69 | 3034.19 | 3537.56 |
| 626.37 | 1156.64 | 1809.87 | 3051.18 | 3580.99 |
| 643.36 | 1168.69 | 1823.41 | 3051.22 | 3593.53 |
| 657.41 | 1188.60 | 1835.96 | 3058.49 | 3613.94 |
| 667.43 | 1208.71 | 1875.05 | 3062.26 | 3635.50 |
| 674.34 | 1244.60 | 1882.05 | 3067.19 | 3682.56 |
| 678.80 | 1256.57 | 1887.10 | 3075.18 | 3700.58 |
| 700.56 | 1267.69 | 1893.02 | 3082.17 | 3706.53 |
| 706.38 | 1274.43 | 1914.12 | 3084.22 | 3735.72 |
| 727.32 | 1280.58 | 1935.03 | 3084.22 | 3736.48 |
| 727.32 | 1283.81 | 1955.13 | 3095.15 | 3749.73 |
| 756.44 | 1296.75 | 1977.97 | 3105.14 | 3770.72 |
| 759.36 | 1299.80 | 1984.96 | 3109.22 | 3789.66 |
| 788.46 | 1317.64 | 1987.94 | 3109.23 | 3809.65 |
| 795.44 | 1336.53 | 1998.98 | 3110.22 | 3826.66 |
| 804.48 | 1350.74 | 2008.93 | 3115.24 | 3845.61 |
| 808.49 | 1364.76 | 2010.01 | 3137.22 | 3863.58 |
| 810.37 | 1381.65 | 2013.87 | 3146.27 | 3901.79 |
| 815.48 | 1403.63 | 2022.92 | 3153.72 | 3902.80 |
| 826.50 | 1432.76 | 2039.39 | 3164.28 | 3920.14 |
| 836.50 | 1442.65 | 2081.08 | 3165.26 | 3924.58 |
| 842.53 | 1446.68 | 2101.10 | 3169.16 | 3957.86 |
| 850.50 | 1452.74 | 2115.01 | 3170.17 | 3975.79 |
| 860.43 | 1459.81 | 2123.09 | 3178.30 | 3981.81 |
| 866.48 | 1480.72 | 2124.03 | 3180.28 | 3982.76 |
| 872.51 | 1482.68 | 2148.02 | 3184.64 | 4061.85 |
| 885.49 | 1490.77 | 2185.64 | 3186.25 | 4070.85 |
| 888.23 | 1496.73 | 2222.30 | 3187.26 | 4081.46 |
| 893.47 | 1500.61 | 2229.13 | 3189.17 | 4084.90 |
| 895.52 | 1509.76 | 2236.17 | 3212.43 | 4090.84 |
| 913.58 | 1515.81 | 2355.19 | 3218.20 | 4105.84 |
| 914.50 | 1522.61 | 2447.33 | 3219.13 | 4115.72 |
| 927.78 | 1524.78 | 2460.20 | 3234.19 | 4120.84 |
| 931.50 | 1576.89 | 2468.27 | 3283.15 | 4134.87 |
| 935.52 | 1585.89 | 2486.28 | 3330.39 | 4135.28 |
| 936.55 | 1592.87 | 2522.20 | 3380.53 | 4151.87 |
| 938.43 | 1642.74 | 2528.65 | 3396.52 | 4159.75 |
| 943.58 | 1652.96 | 2650.17 | 3406.30 | 4183.00 |

**Table (S2). Cont.**: Monoisotopic molecular masses (MM) of components in the ***B. leptochelys*** venom detected by LC/MS analysis.

| **MM** | **MM** | **MM** | **MM** | **MM** |
| --- | --- | --- | --- | --- |
| 4183.01 | 4501.82 | 6843.71 | 7551.33 | 8422.79 |
| 4183.03 | 4524.98 | 6936.86 | 7566.37 | 8447.66 |
| 4192.91 | 4543.12 | 6953.09 | 7586.44 | 8458.92 |
| 4200.02 | 4555.84 | 6974.91 | 7600.20 | 8477.74 |
| 4200.97 | 4579.97 | 6979.21 | 7632.52 | 8522.82 |
| 4206.68 | 4636.98 | 6997.08 | 7646.93 | 8561.51 |
| 4216.04 | 4662.01 | 7004.85 | 7651.61 | 8584.84 |
| 4231.65 | 4738.79 | 7032.93 | 7655.20 | 8613.17 |
| 4239.68 | 4770.04 | 7051.49 | 7764.47 | 10663.59 |
| 4260.26 | 4800.06 | 7090.22 | 7767.57 | 10718.52 |
| 4267.90 | 4803.19 | 7107.38 | 7773.54 | 11050.67 |
| 4288.79 | 4850.09 | 7115.13 | 7784.57 | 11110.46 |
| 4291.90 | 5145.19 | 7127.36 | 7811.53 | 11165.70 |
| 4310.05 | 5218.46 | 7132.24 | 7828.40 | 11209.12 |
| 4312.98 | 5245.51 | 7147.23 | 7846.53 | 11269.36 |
| 4328.97 | 5373.51 | 7161.82 | 7851.47 | 11841.45 |
| 4345.07 | 5447.14 | 7174.74 | 7883.53 | 12042.20 |
| 4346.98 | 5461.95 | 7203.25 | 7935.21 | 12058.19 |
| 4347.86 | 5474.56 | 7221.01 | 7948.23 | 12083.64 |
| 4362.93 | 5528.48 | 7258.17 | 7962.53 | 14051.63 |
| 4363.00 | 5543.84 | 7337.80 | 7974.80 | 14143.31 |
| 4366.89 | 5554.61 | 7343.33 | 7991.40 | 14145.06 |
| 4393.27 | 5637.62 | 7365.13 | 8007.59 | 14232.92 |
| 4419.85 | 5769.99 | 7374.32 | 8027.19 | 14350.08 |
| 4424.82 | 6030.76 | 7398.15 | 8037.42 | 15622.13 |
| 4426.96 | 6237.93 | 7417.79 | 8046.83 | 15659.94 |
| 4432.42 | 6329.54 | 7420.04 | 8070.56 | 15714.91 |
| 4439.86 | 6698.30 | 7428.26 | 8146.12 | 15986.06 |
| 4450.92 | 6723.99 | 7445.65 | 8153.84 |  |
| 4475.02 | 6756.28 | 7458.35 | 8187.64 |  |
| 4480.96 | 6819.73 | 7482.80 | 8214.61 |  |
| 4486.93 | 6834.35 | 7516.40 | 8233.92 |  |

**Table (S3)**: Estimation of the number of disulfide bridges included in each component in the ***B. leptochelys*** venom.

| **Native** | **Alkylated** | **Mass difference** | **NO. of S-S** |  |
| --- | --- | --- | --- | --- |
| 1336.5184 | 1452.7276 | -116.209 | 1 | -0.1512 |
| 1364.7426 | 1480.7087 | -115.966 | 1 | 0.0919 |
| 2906.2945 | 3022.4473 | -116.153 | 1 | -0.0948 |
| 3924.5627 | 4040.6761 | -116.113 | 1 | -0.0554 |
| 3982.7395 | 4098.875 | -116.136 | 1 | -0.0775 |
| 4393.2315 | 4509.9337 | -116.702 | 1 | -0.6442 |
| 4501.7827 | 4617.0411 | -115.258 | 1 | 0.7996 |
| 4579.9345 | 4696.0108 | -116.076 | 1 | -0.0183 |
| 7051.4485 | 7167.4132 | -115.965 | 1 | 0.0933 |
| 7090.1628 | 7206.3796 | -116.217 | 1 | -0.1588 |
| 7343.2603 | 7459.8453 | -116.585 | 1 | -0.527 |
| 7551.2794 | 7667.3727 | -116.093 | 1 | -0.0353 |
| 7948.1787 | 8064.9436 | -116.765 | 1 | -0.7069 |
| 8233.874 | 8349.7353 | -115.861 | 1 | 0.1967 |
| 8477.6931 | 8593.8137 | -116.121 | 1 | -0.0626 |
| 544.32172 | 776.12312 | -231.801 | 2 | 0.3146 |
| 1576.8707 | 1808.9108 | -232.04 | 2 | 0.0759 |
| 3789.6406 | 4021.605 | -231.964 | 2 | 0.1516 |
| 4200.934 | 4433.0442 | -232.11 | 2 | 0.0058 |
| 4206.6513 | 4438.9768 | -232.326 | 2 | -0.2095 |
| 4291.8719 | 4520.9727 | -229.101 | 2 | 3.0152 |
| 4770.0062 | 5002.1449 | -232.139 | 2 | -0.0227 |
| 6974.8397 | 7206.3796 | -231.54 | 2 | 0.5761 |
| 7417.7434 | 7649.1634 | -231.42 | 2 | 0.696 |
| 7551.2794 | 7783.6924 | -232.413 | 2 | -0.297 |
| 8007.5417 | 8239.6658 | -232.124 | 2 | -0.0081 |
| 529.31068 | 878.17283 | -348.862 | 3 | -0.68815 |
| 1403.6171 | 1751.8869 | -348.27 | 3 | -0.0958 |
| 3034.1728 | 3382.3508 | -348.178 | 3 | -0.004 |
| 3051.1598 | 3399.3495 | -348.19 | 3 | -0.0157 |
| 3082.1421 | 3430.3243 | -348.182 | 3 | -0.0082 |
| 3105.1127 | 3453.3951 | -348.282 | 3 | -0.1084 |
| 3164.2545 | 3512.4235 | -348.169 | 3 | 0.005 |
| 3180.249 | 3528.4164 | -348.167 | 3 | 0.0066 |
| 3406.2712 | 3754.4529 | -348.182 | 3 | -0.0077 |
| 3422.5401 | 3770.4604 | -347.92 | 3 | 0.2537 |
| 3580.9641 | 3928.6471 | -347.683 | 3 | 0.491 |
| 3635.4819 | 3983.6581 | -348.176 | 3 | -0.0022 |
| 3682.5407 | 4030.7005 | -348.16 | 3 | 0.0142 |
| 3735.7007 | 4083.8501 | -348.149 | 3 | 0.0246 |
| 3809.6292 | 4157.7879 | -348.159 | 3 | 0.0153 |
| 3982.7395 | 4330.8998 | -348.16 | 3 | 0.0137 |
| 4061.8169 | 4409.693 | -347.876 | 3 | 0.2979 |
| 4081.4323 | 4429.5185 | -348.086 | 3 | 0.0878 |
| 4084.8571 | 4433.0442 | -348.187 | 3 | -0.0131 |
| 4090.8019 | 4438.9768 | -348.175 | 3 | -0.0009 |
| 4105.8089 | 4453.9835 | -348.175 | 3 | -0.0006 |

**Table (S3). Cont.**: Estimation of the number of disulfide bridges included in each component in the ***B. leptochelys*** venom.

| **Native** | **Alkylated** | **Mass difference** | **NO. of S-S** |  |
| --- | --- | --- | --- | --- |
| 4192.8767 | 4541.0619 | -348.185 | 3 | -0.0112 |
| 4199.9684 | 4547.8637 | -347.895 | 3 | 0.2787 |
| 4206.6513 | 4554.8113 | -348.16 | 3 | 0.014 |
| 4239.6442 | 4587.1176 | -347.473 | 3 | 0.7006 |
| 4260.2381 | 4608.7365 | -348.498 | 3 | -0.3244 |
| 4267.8699 | 4616.0378 | -348.168 | 3 | 0.0061 |
| 4291.8719 | 4640.0488 | -348.177 | 3 | -0.0029 |
| 4310.0069 | 4658.1852 | -348.178 | 3 | -0.0041 |
| 4347.8251 | 4696.1365 | -348.311 | 3 | -0.1374 |
| 4419.8286 | 4768.0236 | -348.195 | 3 | -0.021 |
| 4501.7827 | 4850.0569 | -348.274 | 3 | -0.1002 |
| 4636.9595 | 4985.1506 | -348.191 | 3 | -0.0171 |
| 6030.7171 | 6378.8608 | -348.144 | 3 | 0.0303 |
| 6756.236 | 7104.4042 | -348.168 | 3 | 0.0058 |
| 6819.6827 | 7167.4132 | -347.731 | 3 | 0.4435 |
| 6843.6554 | 7191.6721 | -348.017 | 3 | 0.1573 |
| 7051.4485 | 7399.1448 | -347.696 | 3 | 0.4777 |
| 8146.0705 | 8494.309 | -348.238 | 3 | -0.0645 |
| 8613.1147 | 8961.9723 | -348.858 | 3 | -0.6836 |
| 808.48008 | 1272.7074 | -464.227 | 4 | 0.00468 |
| 895.51147 | 1359.7062 | -464.195 | 4 | 0.03727 |
| 938.41936 | 1402.7102 | -464.291 | 4 | -0.05884 |
| 4084.8571 | 4549.1987 | -464.342 | 4 | -0.1096 |
| 4159.7214 | 4624.0446 | -464.323 | 4 | -0.0912 |
| 4231.611 | 4696.0108 | -464.4 | 4 | -0.1678 |
| 5218.4232 | 5682.6176 | -464.194 | 4 | 0.0376 |
| 5245.4714 | 5709.6785 | -464.207 | 4 | 0.0249 |
| 5373.4672 | 5838.1751 | -464.708 | 4 | -0.4759 |
| 5447.1023 | 5911.7495 | -464.647 | 4 | -0.4152 |
| 5637.5809 | 6101.7725 | -464.192 | 4 | 0.0404 |
| 6979.1724 | 7443.347 | -464.175 | 4 | 0.0574 |
| 7090.1628 | 7554.7388 | -464.576 | 4 | -0.344 |
| 7107.3361 | 7571.5579 | -464.222 | 4 | 0.0102 |
| 7203.2076 | 7667.3727 | -464.165 | 4 | 0.0669 |
| 7343.2603 | 7807.8104 | -464.55 | 4 | -0.3181 |
| 7374.2708 | 7839.1445 | -464.874 | 4 | -0.6417 |
| 7586.3885 | 8050.5917 | -464.203 | 4 | 0.0288 |
| 7600.1579 | 8064.9436 | -464.786 | 4 | -0.5537 |
| 7646.8881 | 8110.5908 | -463.703 | 4 | 0.5293 |
| 7784.5262 | 8248.7206 | -464.194 | 4 | 0.0376 |
| 7828.3473 | 8292.6671 | -464.32 | 4 | -0.0878 |
| 7935.1568 | 8399.6916 | -464.535 | 4 | -0.3028 |
| 7948.1787 | 8412.6708 | -464.492 | 4 | -0.2601 |
| 7974.7554 | 8438.6727 | -463.917 | 4 | 0.3147 |
| 7991.3565 | 8455.7155 | -464.359 | 4 | -0.127 |
| 8146.0705 | 8610.8404 | -464.77 | 4 | -0.5379 |
| 8458.8613 | 8922.9968 | -464.136 | 4 | 0.0965 |

**Table (S4)**: Monoisotopic molecular masses (MM) of components in the ***S. m. palmatus*** venom detected by LC/MS analysis.

| **MM** | **MM** | **MM** | **MM** | **MM** |
| --- | --- | --- | --- | --- |
| 516.29 | 771.32 | 1064.53 | 1441.80 | 2089.14 |
| 518.25 | 772.45 | 1071.60 | 1444.81 | 2096.68 |
| 524.30 | 776.45 | 1089.02 | 1480.71 | 2107.99 |
| 524.30 | 788.37 | 1091.52 | 1484.69 | 2110.27 |
| 534.29 | 793.39 | 1093.59 | 1497.83 | 2182.11 |
| 541.19 | 802.48 | 1098.39 | 1503.83 | 2199.98 |
| 549.19 | 811.88 | 1100.58 | 1512.58 | 2229.14 |
| 559.26 | 814.21 | 1103.48 | 1536.67 | 2229.17 |
| 571.26 | 820.39 | 1118.60 | 1540.92 | 2239.29 |
| 571.37 | 826.44 | 1124.50 | 1568.89 | 2245.21 |
| 574.30 | 830.47 | 1125.53 | 1581.43 | 2257.26 |
| 576.30 | 832.39 | 1131.63 | 1592.83 | 2286.24 |
| 584.29 | 843.49 | 1132.59 | 1612.80 | 2300.26 |
| 596.28 | 845.45 | 1145.64 | 1625.92 | 2301.96 |
| 600.33 | 851.38 | 1154.65 | 1649.96 | 2302.23 |
| 603.27 | 853.50 | 1160.50 | 1657.80 | 2310.20 |
| 609.27 | 857.48 | 1171.62 | 1669.91 | 2329.30 |
| 623.29 | 858.43 | 1172.53 | 1711.73 | 2340.16 |
| 645.34 | 873.48 | 1177.66 | 1714.83 | 2341.28 |
| 649.32 | 874.46 | 1180.04 | 1721.00 | 2392.33 |
| 649.32 | 884.51 | 1191.64 | 1731.86 | 2409.36 |
| 656.64 | 886.95 | 1202.66 | 1738.79 | 2448.34 |
| 658.36 | 889.88 | 1204.61 | 1754.03 | 2462.38 |
| 659.33 | 900.43 | 1230.71 | 1768.75 | 2467.41 |
| 660.50 | 902.47 | 1259.68 | 1778.85 | 2475.37 |
| 661.29 | 907.68 | 1282.71 | 1796.99 | 2480.40 |
| 662.65 | 920.47 | 1284.72 | 1804.94 | 2529.22 |
| 666.17 | 924.54 | 1292.69 | 1816.00 | 2536.30 |
| 683.20 | 929.47 | 1299.73 | 1857.28 | 2539.10 |
| 711.23 | 930.64 | 1318.69 | 1868.07 | 2557.43 |
| 717.33 | 931.49 | 1321.68 | 1869.03 | 2622.05 |
| 719.82 | 950.42 | 1359.60 | 1880.94 | 2630.59 |
| 732.29 | 955.38 | 1364.71 | 1912.10 | 2641.42 |
| 735.89 | 956.40 | 1374.77 | 1944.93 | 2642.08 |
| 740.19 | 958.513 | 1381.58 | 1963.20 | 2653.50 |
| 744.17 | 971.54 | 1387.78 | 1968.90 | 2663.06 |
| 745.44 | 976.38 | 1398.69 | 1998.86 | 2695.01 |
| 747.39 | 986.54 | 1399.78 | 2016.08 | 2778.36 |
| 748.40 | 1004.52 | 1404.78 | 2020.95 | 2832.57 |
| 762.44 | 1006.54 | 1408.78 | 2038.16 | 2845.57 |
| 763.91 | 1014.40 | 1421.64 | 2043.14 | 2874.60 |
| 766.08 | 1033.55 | 1421.77 | 2058.03 | 2878.54 |
| 768.48 | 1042.55 | 1421.78 | 2069.12 | 2916.16 |
| 768.48 | 1044.57 | 1430.77 | 2073.53 | 2918.63 |
| 770.46 | 1047.57 | 1431.79 | 2086.39 | 2929.17 |

**Table (S4). Cont.**: Monoisotopic molecular masses (MM) of components in the ***S. m. palmatus*** venom detected by LC/MS analysis.

| **MM** | **MM** | **MM** | **MM** | **MM** |
| --- | --- | --- | --- | --- |
| 2932.14 | 3739.86 | 4753.04 | 6363.79 | 12070.94 |
| 2958.43 | 3753.94 | 4766.16 | 6403.72 | 12179.29 |
| 2959.34 | 3791.00 | 4792.71 | 6492.60 | 12259.02 |
| 2970.65 | 3806.86 | 4806.22 | 6512.86 | 12341.24 |
| 2975.65 | 3817.56 | 4813.87 | 6562.61 | 12354.97 |
| 2975.70 | 3824.98 | 4851.65 | 6579.63 | 12409.80 |
| 3006.51 | 3839.88 | 4876.23 | 6589.62 | 12510.29 |
| 3027.67 | 3855.53 | 4893.59 | 6616.19 | 12518.25 |
| 3088.73 | 3869.88 | 4905.54 | 6750.04 | 13371.87 |
| 3145.77 | 3878.89 | 4919.31 | 7028.62 | 13409.88 |
| 3148.75 | 3892.79 | 4931.14 | 7098.68 | 13430.89 |
| 3166.76 | 3910.12 | 4943.16 | 7864.83 | 13594.95 |
| 3201.82 | 3916.00 | 4959.22 | 8374.94 | 13611.94 |
| 3237.79 | 3924.58 | 4973.22 | 8391.95 | 13652.86 |
| 3245.82 | 3938.61 | 4988.21 | 8445.87 | 13789.17 |
| 3258.84 | 3987.04 | 4991.31 | 8524.05 | 13808.19 |
| 3272.85 | 3992.52 | 5004.33 | 8552.99 | 13856.02 |
| 3282.80 | 4001.94 | 5015.17 | 8745.09 | 13912.17 |
| 3291.72 | 4017.25 | 5032.68 | 8799.01 | 13925.57 |
| 3311.40 | 4043.15 | 5068.34 | 8845.15 | 13947.11 |
| 3315.86 | 4081.90 | 5082.36 | 8858.18 | 13962.28 |
| 3329.87 | 4098.23 | 5105.38 | 8950.94 | 13967.14 |
| 3365.32 | 4143.00 | 5120.17 | 9416.74 | 13993.22 |
| 3387.71 | 4277.38 | 5141.37 | 10268.34 | 14005.18 |
| 3391.36 | 4295.39 | 5155.05 | 10323.43 | 14098.27 |
| 3399.85 | 4310.40 | 5169.65 | 10469.12 | 14117.27 |
| 3401.68 | 4327.33 | 5225.47 | 10649.19 | 14164.3 |
| 3414.84 | 4366.64 | 5264.82 | 11120.26 | 14270.33 |
| 3420.88 | 4396.01 | 5268.43 | 11385.47 | 14301.82 |
| 3473.05 | 4408.06 | 5283.48 | 11404.54 | 14474.75 |
| 3521.55 | 4509.09 | 5300.79 | 11439.42 | 14504.42 |
| 3535.92 | 4546.55 | 5342.40 | 11649.27 | 14676.48 |
| 3547.03 | 4564.87 | 5368.14 | 11683.04 | 15025.62 |
| 3559.47 | 4636.05 | 5380.79 | 11737.35 | 15186.65 |
| 3586.03 | 4651.58 | 5394.51 | 11899.00 | 15351.24 |
| 3643.63 | 4666.58 | 5412.42 | 11959.48 |  |
| 3654.56 | 4675.03 | 5504.45 | 11975.01 |  |
| 3661.96 | 4689.51 | 6244.76 | 12003.52 |  |
| 3698.04 | 4705.23 | 6262.77 | 12015.83 |  |
| 3719.97 | 4745.43 | 6349.80 | 12033.033 |  |

**Table (S5)**: Estimation of the number of disulfide bridges included in each component in the ***S. m. palmatus*** venom.

| **Native** | **Alkylated** | **Mass difference** | **NO. of S-S** |  |
| --- | --- | --- | --- | --- |
| 541.18265 | 657.10083 | -115.918 | 1 | 0.13982 |
| 549.19161 | 665.12374 | -115.932 | 1 | 0.12587 |
| 717.32891 | 833.46322 | -116.134 | 1 | -0.07631 |
| 740.18788 | 856.52575 | -116.338 | 1 | -0.27987 |
| 762.42742 | 878.17241 | -115.745 | 1 | 0.31301 |
| 3166.7264 | 3282.0929 | -115.367 | 1 | 0.6915 |
| 3282.7745 | 3398.9022 | -116.128 | 1 | -0.0697 |
| 3924.5501 | 4040.9282 | -116.378 | 1 | -0.3201 |
| 4905.5023 | 5021.2771 | -115.775 | 1 | 0.2832 |
| 924.52679 | 1156.5413 | -232.0145 | 2 | 0.10149 |
| 1857.2615 | 2089.1191 | -231.8576 | 2 | 0.2584 |
| 2301.9453 | 2534.0522 | -232.1069 | 2 | 0.0091 |
| 2529.1942 | 2761.3032 | -232.109 | 2 | 0.007 |
| 2539.0703 | 2771.1858 | -232.1155 | 2 | 0.0005 |
| 2622.0342 | 2854.1484 | -232.1142 | 2 | 0.0018 |
| 2642.0653 | 2874.1296 | -232.0643 | 2 | 0.0517 |
| 2694.983 | 2927.0989 | -232.1159 | 2 | 0.0001 |
| 2878.5189 | 3110.322 | -231.8031 | 2 | 0.3129 |
| 3166.7264 | 3398.9022 | -232.1758 | 2 | -0.0598 |
| 3311.371 | 3543.4902 | -232.1192 | 2 | -0.0032 |
| 3365.2836 | 3597.3902 | -232.1066 | 2 | 0.0094 |
| 3391.3268 | 3623.4437 | -232.1169 | 2 | -0.0009 |
| 3559.4418 | 3791.792 | -232.3502 | 2 | -0.2342 |
| 3753.9072 | 3986.016 | -232.1088 | 2 | 0.0072 |
| 3817.5292 | 4049.6313 | -232.1021 | 2 | 0.0139 |
| 3824.9448 | 4057.0712 | -232.1264 | 2 | -0.0104 |
| 3878.8638 | 4110.8051 | -231.9413 | 2 | 0.1747 |
| 3915.9578 | 4148.0617 | -232.1039 | 2 | 0.0121 |
| 3986.9984 | 4219.0978 | -232.0994 | 2 | 0.0166 |
| 4792.6691 | 5024.2841 | -231.615 | 2 | 0.501 |
| 5120.1343 | 5352.4269 | -232.2926 | 2 | -0.1766 |
| 6403.6798 | 6635.2008 | -231.521 | 2 | 0.595 |
| 770.45251 | 1118.6007 | -348.1482 | 3 | 0.02581 |
| 1093.5768 | 1441.783 | -348.2062 | 3 | -0.0322 |
| 2622.0342 | 2970.6087 | -348.5745 | 3 | -0.4005 |
| 2630.5676 | 2978.7738 | -348.2062 | 3 | -0.0322 |
| 3753.9072 | 4101.7468 | -347.8396 | 3 | 0.3344 |
| 3839.8518 | 4188.0237 | -348.1719 | 3 | 0.0021 |
| 3938.5816 | 4287.0848 | -348.5032 | 3 | -0.3292 |
| 4327.2881 | 4675.4608 | -348.1727 | 3 | 0.0013 |
| 4366.5969 | 4714.9848 | -348.3879 | 3 | -0.2139 |
| 4745.4023 | 5093.3268 | -347.9245 | 3 | 0.2495 |
| 4876.1964 | 5224.5348 | -348.3384 | 3 | -0.1644 |
| 4905.5023 | 5253.1933 | -347.691 | 3 | 0.483 |
| 5004.2842 | 5352.4269 | -348.1427 | 3 | 0.0313 |

**Table (S5). Cont.**: Estimation of the number of disulfide bridges included in each component in the ***S. m. palmatus*** venom.

| **Native** | **Alkylated** | **Mass difference** | **NO. of S-S** |  |
| --- | --- | --- | --- | --- |
| 5032.6436 | 5380.8926 | -348.249 | 3 | -0.075 |
| 5141.3401 | 5489.4941 | -348.154 | 3 | 0.02 |
| 5169.6114 | 5517.5282 | -347.9168 | 3 | 0.2572 |
| 5225.4301 | 5573.9743 | -348.5442 | 3 | -0.3702 |
| 5268.3994 | 5616.8384 | -348.439 | 3 | -0.265 |
| 5283.4485 | 5632.1186 | -348.6701 | 3 | -0.4961 |
| 5342.3591 | 5690.5368 | -348.1777 | 3 | -0.0037 |
| 5368.0992 | 5716.7309 | -348.6317 | 3 | -0.4577 |
| 6262.7231 | 6610.8624 | -348.1393 | 3 | 0.0347 |
| 6349.7619 | 6697.9027 | -348.1408 | 3 | 0.0332 |
| 6403.6798 | 6751.4785 | -347.7987 | 3 | 0.3753 |
| 6512.8186 | 6860.4343 | -347.6157 | 3 | 0.5583 |
| 661.28587 | 1125.5092 | -464.2233 | 4 | 0.00867 |
| 1091.5116 | 1555.8323 | -464.3207 | 4 | -0.0887 |
| 2069.1009 | 2534.0522 | -464.9513 | 4 | -0.7193 |
| 2409.3344 | 2874.1296 | -464.7952 | 4 | -0.5632 |
| 2462.3517 | 2927.0989 | -464.7472 | 4 | -0.5152 |
| 2480.3718 | 2945.0076 | -464.6358 | 4 | -0.4038 |
| 3521.5168 | 3986.016 | -464.4992 | 4 | -0.2672 |
| 3559.4418 | 3398.9022 | 160.5396 | 4 | 624.7716 |
| 5283.4485 | 5747.552 | -464.1035 | 4 | 0.1285 |
| 8391.9035 | 8856.1153 | -464.2118 | 4 | 0.0202 |
| 8445.8233 | 8910.0393 | -464.216 | 4 | 0.016 |
| 8523.9999 | 8988.1432 | -464.1433 | 4 | 0.0887 |
| 8445.823 | 8910.039 | -464.216 | 4 | 0.016 |
| 8524 | 8988.143 | -464.1433 | 4 | 0.0887 |

**Table (S6)**: Monoisotopic molecular masses (MM) of components in the ***L. quinquestriatus*** venom detected by LC/MS analysis.

| **MM** | **MM** | **MM** | **MM** | **MM** |
| --- | --- | --- | --- | --- |
| 506.28 | 1098.50 | 1726.05 | 2960.18 | 3845.70 |
| 510.32 | 1105.58 | 1749.67 | 2976.18 | 3848.77 |
| 530.27 | 1114.61 | 1773.66 | 2982.16 | 3850.10 |
| 546.29 | 1115.51 | 1786.94 | 2984.15 | 3861.55 |
| 550.28 | 1126.38 | 1791.70 | 2991.23 | 3874.46 |
| 565.25 | 1128.62 | 1796.65 | 3013.43 | 3911.83 |
| 592.15 | 1154.58 | 1800.07 | 3016.74 | 3982.46 |
| 619.36 | 1160.70 | 1802.58 | 3030.10 | 4000.79 |
| 640.29 | 1174.74 | 1812.62 | 3081.20 | 4012.28 |
| 675.88 | 1199.65 | 1828.59 | 3105.22 | 4063.88 |
| 684.42 | 1202.70 | 1852.56 | 3127.20 | 4065.83 |
| 685.33 | 1214.70 | 1882.52 | 3191.19 | 4087.91 |
| 689.42 | 1220.70 | 1901.12 | 3194.30 | 4090.87 |
| 714.32 | 1229.68 | 1906.06 | 3245.12 | 4097.93 |
| 762.40 | 1249.59 | 1906.79 | 3248.20 | 4109.89 |
| 763.41 | 1250.25 | 1948.07 | 3286.36 | 4117.80 |
| 763.42 | 1261.59 | 1954.18 | 3319.35 | 4120.67 |
| 770.42 | 1285.79 | 1955.13 | 3356.34 | 4130.73 |
| 774.39 | 1291.71 | 1959.98 | 3411.63 | 4141.83 |
| 775.40 | 1314.78 | 1996.13 | 3418.43 | 4154.89 |
| 776.46 | 1317.64 | 2007.11 | 3437.31 | 4189.96 |
| 788.46 | 1344.79 | 2011.08 | 3449.48 | 4227.68 |
| 804.48 | 1356.79 | 2013.21 | 3454.62 | 4244.71 |
| 809.78 | 1374.54 | 2017.02 | 3483.46 | 4271.94 |
| 812.52 | 1376.62 | 2019.85 | 3491.46 | 4276.82 |
| 825.46 | 1380.79 | 2021.82 | 3517.44 | 4287.72 |
| 839.59 | 1383.72 | 2053.01 | 3536.93 | 4305.76 |
| 842.95 | 1397.90 | 2082.38 | 3538.42 | 4311.88 |
| 856.42 | 1402.81 | 2094.15 | 3549.37 | 4317.66 |
| 860.431 | 1420.76 | 2098.07 | 3563.46 | 4319.91 |
| 862.38 | 1434.89 | 2111.16 | 3582.54 | 4323.81 |
| 873.46 | 1490.87 | 2126.28 | 3616.53 | 4340.86 |
| 873.63 | 1491.83 | 2181.90 | 3639.01 | 4357.13 |
| 884.46 | 1528.85 | 2182.31 | 3658.58 | 4398.91 |
| 908.55 | 1543.89 | 2193.193 | 3678.46 | 4400.13 |
| 929.57 | 1555.82 | 2207.23 | 3707.97 | 4409.07 |
| 935.52 | 1567.82 | 2224.88 | 3709.66 | 4435.85 |
| 942.67 | 1576.82 | 2254.01 | 3727.67 | 4459.83 |
| 949.47 | 1585.89 | 2288.06 | 3747.49 | 4474.54 |
| 1004.55 | 1591.80 | 2306.95 | 3771.76 | 4489.28 |
| 1005.54 | 1665.90 | 2336.38 | 3781.59 | 4499.09 |
| 1059.56 | 1668.71 | 2506.49 | 3791.75 | 4511.92 |
| 1076.60 | 1673.85 | 2657.15 | 3803.18 | 4522.04 |
| 1086.60 | 1680.91 | 2744.47 | 3825.50 | 4525.10 |
| 1088.65 | 1694.72 | 2756.71 | 3835.47 | 4546.9422 |

**Table (S6). Cont.**: Monoisotopic molecular masses (MM) of components in the ***L. quinquestriatus*** venom detected by LC/MS analysis.

| **MM** | **MM** | **MM** | **MM** | **MM** |
| --- | --- | --- | --- | --- |
| 4612.74 | 6628.7726 | 7172.23 | 7850.30 | 11179.74 |
| 4778.20 | 6635.79 | 7181.48 | 7865.02 | 11318.98 |
| 5052.71 | 6641.87 | 7201.83 | 7904.83 | 11348.15 |
| 5062.28 | 6659.17 | 7227.47 | 7973.36 | 11376.61 |
| 5217.50 | 6670.97 | 7235.17 | 7975.68 | 11394.31 |
| 5462.23 | 6683.92 | 7241.25 | 7986.13 | 11433.24 |
| 5574.97 | 6702.76 | 7246.23 | 8003.69 | 11561.33 |
| 5657.57 | 6715.99 | 7287.30 | 8027.65 | 11576.32 |
| 6095.84 | 6737.81 | 7310.35 | 8041.06 | 11982.70 |
| 6095.85 | 6759.05 | 7315.52 | 8062.38 | 12292.48 |
| 6154.47 | 6789.28 | 7344.74 | 8092.69 | 12318.19 |
| 6208.38 | 6796.30 | 7391.29 | 8100.11 | 12352.87 |
| 6320.81 | 6852.23 | 7409.99 | 8297.42 | 12366.65 |
| 6328.94 | 6890.43 | 7449.31 | 8446.65 | 12398.15 |
| 6382.06 | 6893.62 | 7462.96 | 8590.70 | 12412.12 |
| 6388.56 | 6920.72 | 7463.21 | 9224.86 | 12435.44 |
| 6449.62 | 6947.53 | 7500.55 | 9941.07 | 12448.96 |
| 6457.02 | 6950.13 | 7519.12 | 10088.68 | 12487.13 |
| 6470.35 | 6990.38 | 7552.44 | 10512.52 | 13582.25 |
| 6520.63 | 7000.39 | 7554.36 | 10562.10 | 14203.98 |
| 6561.80 | 7009.43 | 7570.25 | 10837.27 | 14542.56 |
| 6562.75 | 7045.05 | 7582.41 | 10973.20 | 14557.39 |
| 6568.40 | 7072.13 | 7704.56 | 11001.17 | 14657.44 |
| 6589.80 | 7077.22 | 7721.96 | 11016.22 | 14704.14 |
| 6600.39 | 7147.49 | 7728.52 | 11040.65 | 15704.23 |
| 6625.49 | 7162.61 | 7833.66 | 11070.62 | 16828.70 |

**Table (S7)**: Estimation of the number of disulfide bridges included in each component in the ***L. quinquestriatus*** venom.

| **Native** | **Alkylated** | **Mass difference** | **NO. of S-S** |  |
| --- | --- | --- | --- | --- |
| 684.41697 | 800.27434 | -115.857 | 1 | 0.20063 |
| 762.40089 | 878.1725 | -115.772 | 1 | 0.28639 |
| 763.39901 | 879.47323 | -116.074 | 1 | -0.01622 |
| 1076.5903 | 1192.7779 | -116.188 | 1 | -0.1296 |
| 3517.4101 | 3633.3011 | -115.891 | 1 | 0.167 |
| 3791.7269 | 3907.5111 | -115.784 | 1 | 0.2738 |
| 3848.7453 | 3964.7962 | -116.051 | 1 | 0.0071 |
| 3911.7987 | 4027.5956 | -115.797 | 1 | 0.2611 |
| 4109.8557 | 4225.2142 | -115.359 | 1 | 0.6995 |
| 4244.6838 | 4360.3168 | -115.633 | 1 | 0.425 |
| 4271.9015 | 4387.7566 | -115.855 | 1 | 0.2029 |
| 4340.8194 | 4456.7985 | -115.979 | 1 | 0.0789 |
| 4435.8282 | 4551.7399 | -115.912 | 1 | 0.1463 |
| 4459.8036 | 4575.8609 | -116.057 | 1 | 0.0007 |
| 7181.4374 | 7297.2277 | -115.79 | 1 | 0.2677 |
| 7391.2437 | 7507.3276 | -116.084 | 1 | -0.0259 |
| 7463.1711 | 7579.2982 | -116.127 | 1 | -0.0691 |
| 7986.0971 | 8102.5774 | -116.48 | 1 | -0.4223 |
| 3536.8907 | 3768.7555 | -231.865 | 2 | 0.2512 |
| 3771.7293 | 4003.3185 | -231.589 | 2 | 0.5268 |
| 3825.4707 | 4057.8259 | -232.355 | 2 | -0.2392 |
| 3848.7453 | 4080.9037 | -232.158 | 2 | -0.0424 |
| 3982.4328 | 4214.6953 | -232.263 | 2 | -0.1465 |
| 4109.8557 | 4341.7255 | -231.87 | 2 | 0.2462 |
| 4154.8609 | 4386.878 | -232.017 | 2 | 0.0989 |
| 4400.1019 | 4632.8225 | -232.721 | 2 | -0.6046 |
| 4525.0642 | 4757.2187 | -232.155 | 2 | -0.0385 |
| 6918.3443 | 7151.0468 | -232.703 | 2 | -0.5865 |
| 7241.2032 | 7473.3162 | -232.113 | 2 | 0.003 |
| 7462.9149 | 7694.3526 | -231.438 | 2 | 0.6783 |
| 7519.0695 | 7751.4223 | -232.353 | 2 | -0.2368 |
| 1059.5446 | 1407.6483 | -348.104 | 3 | 0.0703 |
| 2960.1596 | 3308.3354 | -348.176 | 3 | -0.0018 |
| 3194.2648 | 3542.4382 | -348.173 | 3 | 0.0006 |
| 3248.1827 | 3596.5307 | -348.348 | 3 | -0.174 |
| 3319.3222 | 3667.5004 | -348.178 | 3 | -0.0042 |
| 3356.3136 | 3704.4908 | -348.177 | 3 | -0.0032 |
| 3411.5981 | 3759.426 | -347.828 | 3 | 0.3461 |
| 3483.4323 | 3831.618 | -348.186 | 3 | -0.0117 |
| 3517.4101 | 3865.598 | -348.188 | 3 | -0.0139 |
| 3538.3929 | 3886.5699 | -348.177 | 3 | -0.003 |
| 3582.5181 | 3930.6992 | -348.181 | 3 | -0.0071 |
| 3616.4962 | 3964.7962 | -348.3 | 3 | -0.126 |
| 3707.9422 | 4055.5544 | -347.612 | 3 | 0.5618 |
| 3709.6337 | 4057.8259 | -348.192 | 3 | -0.0182 |
| 3727.6441 | 4075.8224 | -348.178 | 3 | -0.0043 |
| 3835.4371 | 4183.8518 | -348.415 | 3 | -0.2407 |
| 3848.7453 | 4196.9672 | -348.415 | 3 | -0.0479 |

**Table (S7). Cont.**: Estimation of the number of disulfide bridges included in each component in the ***L. quinquestriatus*** venom.

| **Native** | **Alkylated** | **Mass difference** | **NO. of S-S** |  |
| --- | --- | --- | --- | --- |
| 3911.7987 | 4260.9736 | -348.222 | 3 | -1.0009 |
| 3982.4328 | 4330.9385 | -349.175 | 3 | -0.3317 |
| 4000.7563 | 4348.9356 | -348.506 | 3 | -0.0053 |
| 4012.252 | 4360.3168 | -348.179 | 3 | 0.1092 |
| 4065.8006 | 4414.0062 | -348.065 | 3 | -0.0316 |
| 4087.8719 | 4435.9363 | -348.206 | 3 | 0.1096 |
| 4109.8557 | 4458.0252 | -348.064 | 3 | 0.0045 |
| 4130.6969 | 4478.8966 | -348.17 | 3 | -0.0257 |
| 4154.8609 | 4502.825 | -348.2 | 3 | 0.2099 |
| 4227.6529 | 4575.8609 | -347.964 | 3 | -0.034 |
| 4317.6351 | 4665.757 | -348.208 | 3 | 0.0521 |
| 4357.088 | 4705.2655 | -348.122 | 3 | -0.0035 |
| 4400.1019 | 4748.2813 | -348.178 | 3 | -0.0054 |
| 4435.8282 | 4783.9793 | -348.179 | 3 | 0.0229 |
| 4511.8807 | 4859.8865 | -348.151 | 3 | 0.1682 |
| 4525.0642 | 4873.2329 | -348.006 | 3 | 0.0053 |
| 6568.3579 | 6916.1767 | -348.169 | 3 | 0.3552 |
| 6890.3984 | 7238.2187 | -347.819 | 3 | 0.3537 |
| 6947.4895 | 7295.6747 | -347.82 | 3 | -0.0112 |
| 7077.1763 | 7425.3561 | -348.185 | 3 | -0.0058 |
| 7172.1916 | 7520.3172 | -348.18 | 3 | 0.0484 |
| 7344.6969 | 7692.3473 | -348.126 | 3 | 0.5236 |
| 862.38178 | 1326.7235 | -464.342 | 4 | -0.10972 |
| 3563.4445 | 4027.5956 | -464.151 | 4 | 0.0809 |
| 3678.4292 | 4142.6873 | -464.258 | 4 | -0.0261 |
| 3982.4328 | 4446.3213 | -463.889 | 4 | 0.3435 |
| 4065.8006 | 4529.8132 | -464.013 | 4 | 0.2194 |
| 4087.8719 | 4551.7399 | -463.868 | 4 | 0.364 |
| 4130.6969 | 4595.1382 | -464.441 | 4 | -0.2093 |
| 4154.8609 | 4618.8541 | -463.993 | 4 | 0.2388 |
| 4357.088 | 4821.9279 | -464.84 | 4 | -0.6079 |
| 4409.0306 | 4873.2329 | -464.202 | 4 | 0.0297 |
| 5217.4704 | 5681.6768 | -464.206 | 4 | 0.0256 |
| 6320.7657 | 6784.7444 | -463.979 | 4 | 0.2533 |
| 6702.7219 | 7166.6081 | -463.886 | 4 | 0.3458 |
| 6789.229 | 7254.0435 | -464.814 | 4 | -0.5825 |
| 6890.3984 | 7354.5625 | -464.164 | 4 | 0.0679 |
| 6990.337 | 7454.2884 | -463.951 | 4 | 0.2806 |
| 7009.3861 | 7473.3162 | -463.93 | 4 | 0.3019 |
| 7241.2032 | 7705.3468 | -464.144 | 4 | 0.0884 |
| 7287.243 | 7751.4223 | -464.179 | 4 | 0.0527 |
| 7409.9454 | 7873.4492 | -463.504 | 4 | 0.7282 |
| 7462.9149 | 7926.4005 | -463.486 | 4 | 0.7464 |
| 7500.5029 | 7964.3585 | -463.856 | 4 | 0.3764 |
| 7519.0695 | 7983.601 | -464.532 | 4 | -0.2995 |
| 7850.244 | 8314.8313 | -464.587 | 4 | -0.3553 |

**Antibacterial activity**

The antibacterial activity of the crude venom samples was tested against *Escherichia coli*, *Bacillus subtilis*, and *Staphylococcus aureus* via the agar diffusion technique. Hydrophobic fractions eluted between 40 and 55 min were also tested, since they contain linear antimicrobial peptides (Fig. S4). The results, as shown in Table 2, reveal minimal antibacterial effects for the examined venoms. Venom from *B. leptochelys* exhibited low activity against *E. coli* at 10 µg and against *B. subtilis* at both 5 and 10 µg. *S. m. palmatus* venom showed slight activity only against *B. subtilis* at both examined concentrations. *L. quinquestriatus* venom displayed marginal activity against both (*E. coli & B. subtilis*), with the strongest effect observed against *E. coli* at 10 µg. None of the crude venoms showed any ability to inhibit the growth of *S. aureus* at the tested doses. Also, none of the hydrophobic fractions from these three venoms, specifically those eluting between 40 and 55 min, showed detectable antibacterial activity under the given assay conditions.

**Table S8.** Antimicrobial activity assay via LB agar plates.

|  | Halo zone diameter (cm) | | | | | | |
| --- | --- | --- | --- | --- | --- | --- | --- |
| Scorpion species | ***E. coli*** | | ***B. subtilis*** | | | ***S. aureus*.** | |
|  | 5 μg | 10 μg | 5 μg | 10 μg | | 5 μg | 10 μg |
| ***B. leptochelys*** | - | 0.5 | 0.1 | | 0.2 | - | - |
| ***S. m. palmatus*** | - | - | 0.1 | | 0.3 | - | - |
| ***L. quinquestriatus*** | 0.1 | 0.2 | 0.1 | | 0.2 | - | - |
